# Estimating the prevalence of visual impairment in the Netherlands, with forecasts up to 2050: a meta-analysis of national databases

**DOI:** 10.1016/j.eclinm.2026.103858

**Published:** 2026-04-01

**Authors:** Ellen B.M. Elsman, T. Petra Rausch-Koster, Hilde P.A. van der Aa, Jeroen Hoogland, Robert P.L. Wisse, H. Susan J Picavet, W.M. Monique Verschuren, Tos T.J.M. Berendschot, Joost W. Vanhommerig, Wishal D. Ramdas, Victor A. de Vries, Martijn Huisman, Tamara Brussee, Cees van der Windt, Marc B. Muijzer, Petra van Es, Jan E.E. Keunen, Caroline C.W. Klaver, Ruth MA. van Nispen

**Affiliations:** a Ophthalmology, Amsterdam UMC, Vrije Universiteit Amsterdam, Amsterdam Public Health research institute, Amsterdam, the Netherlands; b Epidemiology and Data Science, Amsterdam UMC, Vrije Universiteit Amsterdam, Amsterdam, the Netherlands; c Equipe Zorgbedrijven BV, Xpert Clinics Oogzorg, Zeist, the Netherlands; d Centre for Prevention, Lifestyle & Health, National Institute for Public Health and the Environment, Bilthoven, the Netherlands; e University Eye Clinic Maastricht, Maastricht, the Netherlands; f Nivel, Netherlands Institute for Health Services Research, Utrecht, the Netherlands; g Ophthalmology, Erasmus Medical Center, Rotterdam, the Netherlands; h Hogeschool Utrecht, Utrecht, the Netherlands; i Ziekenhuis Rivierenland, Tiel, the Netherlands; j Dutch Healthcare Authority (NZa), Utrecht, the Netherlands; k Bartiméus, Zeist, the Netherlands; l Ophthalmology, Radboud University Medical Center, Nijmegen, the Netherlands

**Keywords:** Visual impairment, Prevalence, Meta-analyses, Blindness, Epidemiology

## Abstract

**Background:**

Current prevalence estimates of visual impairment (i.e., low vision and blindness) in the Netherlands are lacking. Based on representative databases, we aimed to estimate the prevalence of visual impairment among Dutch adults and to forecast estimates to 2050.

**Methods:**

We undertook a meta-analysis of national databases. Databases were primarily identified through expert consultation and included when representative of the Dutch population and containing recent (≥2010) data on vision (visual acuity <6/18, self-reported visual functioning, or relevant International Classification of Primary Care [ICPC] codes). We classified self-reported visual functioning according to four definitions: A) only near vision difficulties; B) only distance vision difficulties; C) both near and distance vision difficulties; D) any vision difficulties (combining A–C). Population-based prevalences were calculated and pooled using four meta-analyses, including heterogeneity assessment and subgroup analyses. Age-effects were modelled using logistic mixed-effects models.

**Findings:**

We included eight databases with data collected data between 2010 and 2024, accounting for 1,814,716 individuals (894,541 males and 920,175 females). Five databases were prospective population-based cohort studies, two were periodically administered cross-sectional health surveys, and one contained registrations from general practices. Five databases had self-reported data on visual functioning, two had data on best-corrected distance visual acuity, and one included ICPC codes. There was substantial heterogeneity with assessment method as moderator (i.e., self-reported vs. visual acuity vs. ICPC; I^2^ statistic >98%, p < 0.0001). The pooled prevalence of visual impairment was 0.28% (95% CI: 0.11–0.73) for visual acuity (2 databases), whereas it was 0.51% (95% CI: 0.50–0.52) for ICPC (1 database). The prevalence for self-report (five databases) depended on the definition. For only near vision difficulties (A), prevalence was 1.81% (95% CI: 1.01–3.21); for only distance vision difficulties (B), prevalence was 0.63% (95% CI: 0.50–0.80); for both near and vision difficulties (C), prevalence was 0.51% (95% CI: 0.31–0.83); and for any vision difficulties (D), prevalence was 3.21% (95% CI: 2.15–4.77). After applying age-specific and assessment-specific prevalence estimates to projected population structures, we estimate that 39,100–406,400 Dutch adults have visual impairment in 2025, and this is estimated to increase to 48,800–489,100 by 2050 owing to population growth and ageing, depending on definitions.

**Interpretation:**

Despite inherent limitations (including databases mostly containing self-reported data on visual functioning) and substantial uncertainty in estimates, this study provides population-level estimates of visual impairment in the Netherlands. Although these estimates span a wide range of values, they reveal trends and plausible uncertainty intervals, illustrating the potential magnitude of the current and future burden of visual impairment.

**Funding:**

Dutch Eye Foundation (Oogfonds).

**Research in context**

**Evidence before this study**

Low vision and blindness are leading causes of disability worldwide, yet prevalence estimates for visual impairment in the Netherlands, a country that currently inhabits 18 million people, are outdated. To estimate the current prevalence of visual impairment among adults in the Netherlands, and forecast the prevalence up to 2050, we undertook a meta-analysis of national representative databases. We identified databases primarily through expert consultation and snowballing among researchers and policy makers in the field of ophthalmology, epidemiology, and public health who are familiar with population-based data sources in the Netherlands. Databases were included if they contained data on vision (i.e., visual acuity, self-reported data on visual functioning, or codes related to vision from the International Classification of Primary Care [ICPC]), had their last measurement in or after 2010, and were population-based and as such representative of the Dutch population. We included data from eight databases (n = 1,814,716). The pooled prevalence of visual impairment ranged from 0.28% to 3.21%, depending on assessment method and definition. We also modelled prevalence data to account for the increasing prevalence of vision impairment with age, resulting in age-specific prevalence estimates.

**Added value of this study**

By using multiple representative databases containing data on a large proportion of the Dutch adult population (12%), our study provides a more current estimate of the prevalence, actual numbers, and future projections of visual impairment in the Netherlands. We estimate that 39,100–406,400 Dutch adults have visual impairment in 2025, which is projected to increase to 48,800–489,100 by 2050 owing to population growth and ageing.

**Implications of all the available evidence**

Our estimates span a wide range of values, reflecting substantial uncertainty, but most are lower than those forecasted in previous studies, despite increased population growth. This difference is potentially caused by new treatment options. However, limitations of our study, including relying primarily on expert consultation to identify databases and most databases containing self-reported data on visual functioning, should be taken into account. Yet, our findings are in line with those of neighbouring countries and suggest a growing need for eye care due to demographic ageing. Uncorrected refractive errors may constitute an important and preventable cause of visual impairment, underscoring the potential benefit of improved access to eye care and vision screening.

## Introduction

Visual impairment, which includes low vision and blindness, is one of the leading causes of disability worldwide.^1^, ^2^, ^3^, ^4^ Globally, 1.1 billion people were living with vision loss in 2020; 43 million were blind (crude prevalence 0.5%) and 295 million people had moderate to severe visual impairment (crude prevalence 3.7%).^5^ In western Europe, the crude prevalence of blindness was 0.4% in 2020, whereas for moderate to severe visual impairment it was 3.5%.^5^ The prevalence increases with older age and is highest among frail elderly and those living in nursing homes.^6^^,^^7^ In western Europe, uncorrected refractive errors and cataract are the leading causes of moderate to severe visual impairment, whereas age-related macular degeneration is the leading cause of blindness.^8^

In the Netherlands (population just over 18 million in 2024),^9^ prevalence estimates are lacking. There is no central designated registry keeping track of the number of people with visual impairment. Current estimates range from 150,000–400,000 people. A study in 2005, based on data from comparable countries, estimated that there were almost 300,000 adults (2.4%) with visual impairment, predicted to increase to over 350,000 in 2020.^6^ However, data from other countries could only partially be used and the predicted population growth was underestimated. Another projection for 2020 was made in 2011, resulting in an expected number of almost 380,000.^10^ Country-specific estimates from the Global Burden of Disease study for 2020 are available too, suggesting 465,000 people have moderate to severe visual impairment (crude prevalence 2.7%).^5^ However, these estimates are based on two outdated studies with subnational coverage.^11^^,^^12^ Moreover, new treatment options for main causes of visual impairment have become available, such as anti-vascular endothelial growth factor (anti-VEGF) injections for macular oedema and retinal damage,^13^ reducing the number of people with visual impairment.^14^

There are, however, representative (cohort) studies that collect all sorts of health information for research purposes, including data on visual parameters (i.e., visual acuity, self-reported visual functioning or primary care classifications). These databases can be used by others, and would allow current estimates of the prevalence of visual impairment and forecasting. Such estimates are needed to support scenario-based public health planning and to inform discussions on future directions for eye care and low vision services. Using raw data on visual parameters collected in representative databases, this study aims to estimate the current prevalence of visual impairment among adults in the Netherlands, and forecast the prevalence up to 2050, taking the future population structure into account.

## Methods

### Study design and ethics

To estimate the current prevalence of visual impairment among adults in the Netherlands, and forecast the prevalence up to 2050, we undertook a meta-analysis of national representative databases. After reviewing the study protocol, the Medical Ethical Committee of Amsterdam UMC, the Netherlands, confirmed that the study protocol did not fall under the Medical Research Involving Human Subjects Act (2023.0336) and was exempt. The included databases were obtained from studies approved by a medical ethical committee in the Netherlands and conducted in accordance with the Declaration of Helsinki. Informed consent was obtained from all participants in these studies. Appendix 1 provides a description of the included databases and their ethical approval. Reporting of the study was in line with applicable items from the STROBE guideline.^15^

### Databases

Relevant databases were identified primarily through expert consultation and snowballing, involving researchers and policy-makers in the field of ophthalmology, epidemiology, and public health who are familiar with population-based data sources in the Netherlands. To complement this approach, we also conducted non-systematic, targeted searches in the scientific literature and on the internet for Dutch population-based health studies. Databases were included if they contained visual parameters, the last measurement was performed in or after 2010, and the sample was population-based and as such broadly representative of the Dutch population. Appendix 2 provides information about the sampling frame, recruitment, and representativeness of included databases. Visual parameters were visual acuity measured with a vision chart, self-reported data on visual functioning, or codes related to vision from the International Classification of Primary Care v1 (ICPC).^16^

### Assessment of visual impairment

#### Visual acuity

We included databases that measured presenting near or distance visual acuity (where visual acuity was measured with the habitual correction a person is using), or best-corrected near or distance visual acuity (where a pinhole or refraction were used). We applied criteria of the World Health Organisation (WHO) to categorise people based on visual acuity in the better-seeing eye:^17^ no visual impairment (visual acuity ≥6/12), mild visual impairment (visual acuity ≥6/18 and <6/12), moderate visual impairment (visual acuity ≥6/60 and <6/18), severe visual impairment (visual acuity ≥3/60 and <6/60), and blindness (visual acuity <3/60).

#### Self-reported visual functioning

For self-reported visual functioning two standardised questions were often used:^18^ “Can you (with glasses/contact lenses if necessary) read the small print of a newspaper?” and “Can you (with glasses/contact lenses if necessary) recognise faces at a distance of 4 m?”. Response options to these questions were: “yes, without difficulty”, “yes, with some difficulty”, “yes, with much difficulty”, and “no, I cannot”. We defined having self-reported visual impairment according to four criteria:^19^

A Much difficulty or inability to read small print (only near vision difficulties)
B Much difficulty or inability to recognise faces (only distance vision difficulties)
C Much difficulty or inability with both task A *and* task B
D Any of the previous

In one database part of the NEI VFQ-25 (National Eye Institute 25-item Vision Function Questionnaire) was administered.^20^ Here, we categorised responses to the questions “How much difficulty do you have reading ordinary print in newspapers?” and “How much difficulty do you have reading street signs or the names of stores?”, and defined the responses “extreme difficulty” and “stopped doing this because of eyesight” as visual impairment, following criteria A–D.

#### ICPC codes

The Nivel Primary Care Database (NPCD) includes electronic health records from Dutch general practices from 2011 onwards. Diagnoses, symptoms, and complaints are coded using the ICPC,^16^ with each health issue typically recorded as an episode of care. GPs register ICPC codes based on the reason for encounter or diagnosis. We selected codes F28 (functional limitations/disabilities of eye/adnexa) and F94 (blindness-any degree/form) as visual impairment, if ever registered for a patient.

### Statistical analyses

References for statistical methods, software and packages are included in Appendix 3.

#### Analyses of separate databases

Data from each separate database were analysed with SPSS version 28. We applied external weighing to make data representative for the Dutch population in terms of sex and age (in 5-year age categories) for the year of data collection (±1 year). Sociodemographic and clinical characteristics were calculated using descriptive statistics. The population-based prevalence was calculated from the raw proportion using the definitions of visual impairment described above, and 95% confidence intervals were calculated using the Wilson method. Stratified prevalences for sex and age categories, including working age (2017 and earlier: 18–65; 2018 or later: 18–66)^21^ were also calculated. Because of external weighing, all prevalence estimates represent age- and sex-standardised estimates.

#### Meta-analyses of proportions

Prevalence estimates from separate databases were pooled using a meta-analysis of proportions in R version 4.0.5, with ‘meta’ and ‘metafor’. For visual acuity, we combined the moderate, severe, and blind categories and defined this as visual impairment.^17^ Separate meta-analyses were performed to account for the four self-report definitions of visual impairment (A–D), which are conceptually different. All databases were analysed jointly within a single modelling framework, under the assumption that different assessment methods capture the same underlying construct of visual impairment but with systematic measurement differences. This approach allows borrowing of information across databases and enables formal evaluation of assessment method as a source of heterogeneity. Consequently, the same visual acuity and ICPC databases appear in each of the four meta-analyses, while self-reported data differ by definition. Because between-study variance is re-estimated within each model, pooled estimates for visual acuity and ICPC may differ slightly across analyses. A logit transformation was performed to address small proportions; robustness of the results was checked using a double arcsine transformation. We used a random effects model to account for within and between-database variances with the DerSimonian and Laird method to pool population-based prevalences. The I^2^ statistic was used to estimate heterogeneity; values >50% indicate substantial heterogeneity. Potential sources of heterogeneity were identified through diagnostic tests for outlying and influential databases (Baujat plot, externally studentised residuals, and leave-one-out diagnostics). Outlying and influential databases were considered for deletion if reasonable. We conducted subgroup analyses to examine the impact of database characteristics (assessment method: self-report vs. visual acuity vs. ICPC; sample size: <10,000 vs. ≥10,000; region: local vs. national; study design: cohort vs. cross-sectional) on the heterogeneity.

#### Sensitivity analysis

To evaluate whether the joint modelling framework influenced pooled estimates for the self-reported outcomes, we conducted a sensitivity analysis restricted to self-report databases only. Because definition C (both near and distance vision difficulties) is the most restrictive self-report definition and yields the lowest prevalence estimates, we selected this definition as a conservative test case in which any influence of borrowing information from visual acuity and ICPC databases would be most apparent. In this sensitivity analysis, the meta-analysis was repeated including only the self-report databases corresponding to definition C, excluding visual acuity and ICPC datasets. The same random-effects meta-analytic approach and logit transformation were applied as in the primary analyses.

#### Modelling age effects

We subsequently modelled prevalence data to account for the increasing prevalence of vision impairment with age using logistic mixed-effects models with ‘lme4’ in R. Again, separate analyses were performed to account for the conceptually different self-report definitions (A–D), whereas data on visual acuity and ICPC codes were identical across the four analyses. We harmonised age groups by calculating the midpoint of each age category and added three years to the lower age bound for open-ended categories (i.e., 85+, 90+, and 95+). The primary fixed effects model included an intercept, a natural spline for age (with two degrees of freedom) to flexibly capture non-linear age-related trends, and assessment method. Random study-level intercepts were included for each study to account for variability across all datasets. Models were compared using likelihood ratio tests to assess the significance of the assessment method. Predicted values and 95% confidence intervals were visualised using ‘ggeffects’ and and ‘ggplot2’. Separate predictions were generated for each assessment method to investigate age-related trends.

#### Projecting the burden of visual impairment

Based on the age- and assessment-specific prevalence estimates from the logistic mixed-effects models, we projected the number of adults with visual impairment for the current year (2025), and at decade increments up to 2050. Here, we applied the predicted prevalence estimates and 95% confidence intervals from the logistic mixed-effects model to the projected population structures, obtained from Statistics Netherlands.^9^ For self-report, we forecasted separate projections to account for the different definitions (A–D). For prevalence estimates based on visual acuity and ICPC codes, we averaged the estimates from the four models. Forecasts assume that the age-specific prevalence rates remain stable over time, and only reflect demographic ageing and population growth, rather than changes in underlying risks or treatments, as well as other epidemiological transitions.

### Role of the funding source

The funder of the study had no role in the study design, data collection, data analysis, data interpretation, or writing of the report.

## Results

**Fig. 1:**
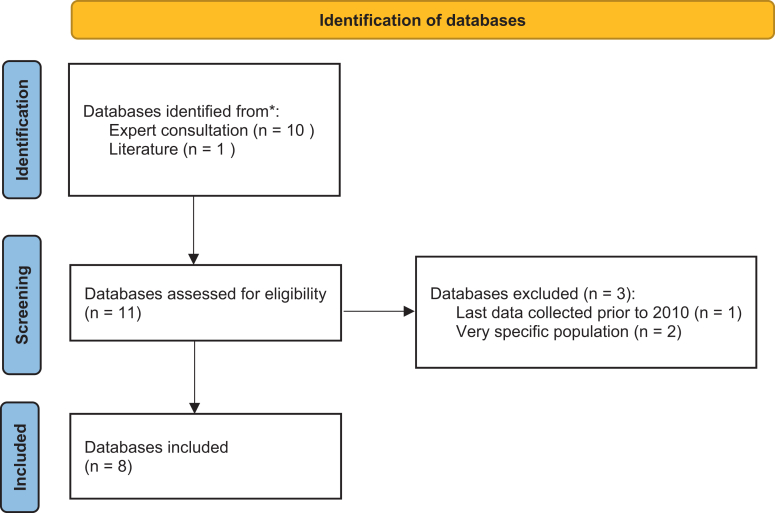
Flow diagram of database selection.

Eleven databases with visual parameters were initially identified; after applying eligibility criteria, eight were included (Fig. 1, Table 1).^22^, ^23^, ^24^, ^25^, ^26^, ^27^, ^28^, ^29^ Excluded databases performed the last measurement before 2010,^30^ or included a very specific population not representative of the Dutch population.^7^^,^^31^ Five included databases were prospective population-based cohort studies,^22^^,^^23^^,^^27^, ^28^, ^29^ two were periodically administered cross-sectional health surveys,^25^^,^^26^ and one contained registrations from general practices.^24^ Five databases had self-reported data on visual functioning,^25^, ^26^, ^27^, ^28^, ^29^ two had data on best-corrected distance visual acuity,^22^^,^^23^ and one included ICPC codes.^24^ Sample sizes ranged from 1047 (LASA)^28^ to 1,240,330 persons (NPCD),^24^ with a combined total of 1,814,716 persons (894,541 males/920,175 females). Appendix 4 contains unweighted characteristics for each database.

**Table 1:** Descriptive characteristics (weighted for age and sex) of the included databases.

Database	Database type	Period of data collection	Type of vision data	Characteristics of study population		
				n	% Female	Age, mean (SD) [range]
The Maastricht Study	Prospective population-based cohort study	2011–2020	Best corrected visual acuity: ETDRS chart	7739	50.3	56.4 (10.2) [40–79]
Rotterdam Study	Prospective population-based cohort study	2010–2020	Best corrected visual acuity: ETDRS chart	7684	51.7	62.0 (11.8) [41–100]
NPCD (Nivel Primary Care Database)	Registration data general practices	2022	ICPC codes	1,240,330	50.7	18–24: 101,434 (8.2)^a^
						25–29: 101,579 (8.2)
						30–34: 102,830 (8.3)
						35–39: 95,980 (7.7)
						40–44: 93,480 (7.5)
						45–49: 97,456 (7.9)
						50–54: 114,881 (9.3)
						55–59: 113,307 (9.1)
						60–64: 103,727 (8.4)
						65–69: 91,086 (7.3)
						70–74: 84,292 (6.8)
						75–79: 63,893 (5.2)
						80–84: 40,966 (3.3)
						85+: 35,418 (2.9)
GEMON (Dutch Public Health Monitor)	Cross-sectional health survey	2020	Self-report: 2 questions	514,967	50.7	50.4 (18.4) [26–75]^b^
GECON (Dutch Health Survey)	Cross-sectional health survey	2020–2022	Self-report: 2 questions	21,917	50.7	50.4 (18.4) [26–75]^b^
Doetinchem Cohort Study	Prospective population-based cohort study	2018–2022	Self-report: 2 questions	2731	51.4	65.3 (9.9) [51–90]
LASA (Longitudinal Ageing Study Amsterdam)	Prospective population-based cohort study	2021–2022	Self-report: 2 questions	1047	52.7	72.6 (7.6) [63–97]
Lifelines	Prospective population-based cohort study	2021–2024	Self-report: NEI VFQ-25^c^	18,301	50.7	50.6 (18.3) [20–96]

ETDRS: Early Treatment Diabetic Retinopathy Study; ICPC: International Classification of Primary Care; NEI VFQ-25: National Eye Institute 25-item Vision Function Questionnaire.

a Data represented as age group, n (%).

b Range represents the 10th^–^90th percentile.

c Not all items from the NEI VFQ-25 were administered, see eye_conditions_oq [Lifelines Wiki] (rug.nl).

Table 2 presents population-based prevalences of visual impairment for each database. Prevalences of moderate to severe visual impairment and blindness based on visual acuity or ICPC codes ranged from 0.2–0.5%. For databases with self-reported data, prevalences varied by definitions. For only near vision difficulties (A), prevalences ranged from 0.5–5.3%, whereas for only distance vision difficulties (B), prevalences ranged from 0.4–1.0%. Prevalences were lowest for both near and distance vision difficulties (C, ranging from 0.2–1.0%) and highest for any vision difficulties (D, ranging from 1.6–6.5%). Appendix 5 presents stratified prevalences of visual impairment for sex and age (including working age) for each database, showing that prevalences increased with age.

**Table 2:** Age- and sex-standardised prevalence of visual impairment per database.

Database	Sample size, n	Cases visual impairment, n	Prevalence visual impairment, % (95% CI)
The Maastricht Study	7739	Mild: 52	Mild: 0.7 (0.5–0.9)
		Moderate: 14	Moderate: 0.2 (0.1–0.3)
		Severe: 0	Severe: 0.0 (0.0–0.0)
		Blind: 0	Blind: 0.0 (0.0–0.0)
		Combined^a^: 14	Combined^a^: 0.2 (0.1–0.3)
Rotterdam Study	7684	Mild: 27	Mild: 0.4 (0.3–0.6)
		Moderate: 28	Moderate: 0.4 (0.3–0.6)
		Severe: 1	Severe: 0.0 (0.0–0.0)
		Blind: 5	Blind: 0.1 (0.1–0.2)
		Combined^a^: 34	Combined^a^: 0.5 (0.3–0.7)
NPCD (Nivel Primary Care Database)	1,240,330	6318	0.5 (0.5–0.5)
GEMON (Dutch Public Health Monitor)^b^	514,967	A: 14,519	A: 2.8 (2.8–2.8)
		B: 4093	B: 0.8 (0.8–0.8)
		C: 5035	C: 1.0 (1.0–1.0)
		D: 23,666	D: 4.6 (4.5–4.7)
GECON (Dutch Health Survey)^b^	21,917	A: 427	A: 1.9 (1.7–2.1)
		B: 123	B: 0.6 (0.5–0.7)
		C: 156	C: 0.7 (0.6–0.8)
		D: 706	D: 3.2 (3.0–3.4)
Doetinchem Cohort Study^b^	2731	A: 146	A: 5.3 (4.5–6.2)
		B: 17	B: 0.6 (0.4–1.0)
		C: 14	C: 0.5 (0.3–0.8)
		D: 177	D: 6.5 (5.6–7.5)
LASA (Longitudinal Ageing Study Amsterdam)^b^	1047	A: 5	A: 0.5 (0.2–1.1)
		B: 11	B: 1.0 (0.6–1.8)
		C: 6	C: 0.6 (0.3–1.3)
		D: 22	D: 2.1 (1.4–3.2)
Lifelines^b^	18,301	A: 178	A: 1.0 (0.9–1.2)
		B: 82	B: 0.4 (0.3–0.5)
		C: 30	C: 0.2 (0.1–0.3)
		D: 290	D: 1.6 (1.4–1.8)

a Combined refers to the combination of moderate, severe and blind; these categories were combined and defined as visual impairment in the meta-analyses.

b Prevalence of visual impairment based on self-reported data is categorised by four definitions: A: only near vision difficulties; B: only distance vision difficulties; C: both near and distance vision difficulties; D: any vision difficulties.

**Fig. 2:**
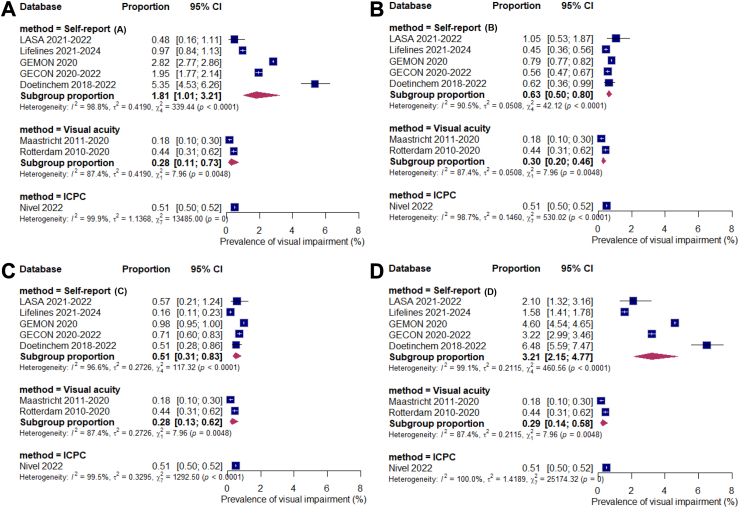
Forest plots of visual impairment prevalence (age- and sex-standardised) stratified by assessment method^a^. ^a^Each panel includes all self-report data for the respective definition, together with identical visual acuity and ICPC (International Classification of Primary Care) data; Even though based on the same data, visual acuity and ICPC estimates and confidence intervals may vary slightly across panels because they were analysed in separate meta-analytic models with a random effects model; Prevalence of visual impairment based on self-reported data is categorised by four definitions: A: only near vision difficulties (upper left); B: only distance vision difficulties (upper right); C: both near and distance vision difficulties (bottom left); D: any vision difficulties (bottom right).

Meta-analyses of proportions revealed substantial heterogeneity across databases (I^2^ statistic >98%, p < 0.0001). Consequently, we performed subgroup analyses for moderating effects. Here, assessment method moderated effects for only near vision difficulties (A, p < 0.0001) and any vision difficulties (D, p < 0.0001), but not for only distance vision difficulties (B, p = 0.050) and both near and distance vision difficulties (C, p = 0.48). Proportions of heterogeneity accounted for (R^2^) were 82.6% and 90.4% for A and D respectively. However, heterogeneity among subgroups remained high (I^2^ statistic visual acuity 87.4%, p = 0.0048; I^2^ statistic self-report >90%, p < 0.0001). Diagnostic tests for outlying and influential databases gave no reasons to exclude databases. Because of the effect of assessment method, Fig. 2 presents the forest plots of visual impairment prevalence per method for the four models. The pooled prevalence of visual impairment for visual acuity was between 0.28–0.30% (95% CI: between 0.11–0.73), whereas prevalence for ICPC codes (one database) was 0.51% (95% CI: 0.50–0.52). The pooled prevalence for self-reported visual functioning ranged from 0.51% (95% CI: 0.31–0.83) to 3.21% (95% CI: 2.15–4.77), depending on the definition. The double arcsine transformation resulted in similar estimates, confirming the robustness of the results (Appendix 6). Results of the sensitivity analysis restricted to self-report databases for definition C were highly similar to those obtained from the joint model (Appendix 7). The pooled prevalence estimate was 0.50% (95% CI 0.28–0.89) compared with 0.51% (0.31–0.83) in the primary analysis that included visual acuity and ICPC datasets. This indicates that inclusion of visual acuity and ICPC data in the joint modelling framework did not materially influence the pooled prevalence estimates.

**Fig. 3:**
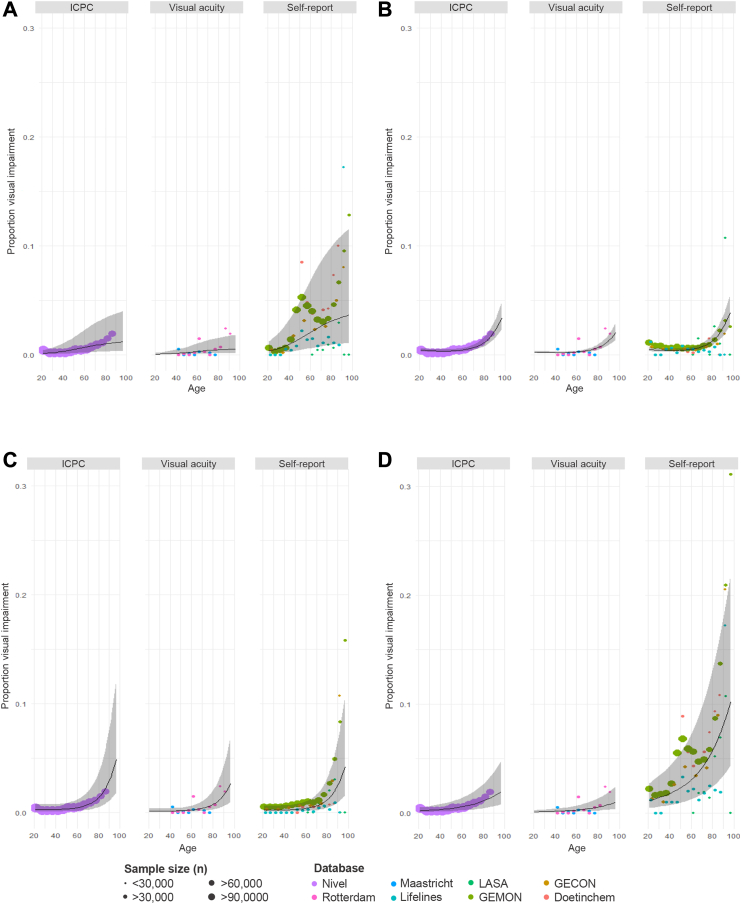
Estimated prevalence (age- and sex-standardised) of visual impairment with age stratified by assessment method^a^. ^a^Each panel includes all self-report data for the respective definition, together with identical visual acuity and ICPC (International Classification of Primary Care) data; Visual acuity and ICPC curves may vary slightly across panels because they were analysed within separate models; Prevalence of visual impairment based on self-reported data is categorised by four definitions: A: only near vision difficulties (upper left); B: only distance vision difficulties (upper right); C: both near and distance vision difficulties (bottom left); D: any vision difficulties (bottom right).

When accounting for the increasing prevalence of vision impairment with age with logistic mixed-effects models, model fit significantly improved in most models after including assessment method compared to a model with age only (A, p = 0.023; B, p = 0.0468; D, p = 0.0007). However, model fit did not improve for both near and distance vision difficulties (C, p = 0.49). Fig. 3 shows the prevalence of visual impairment by age for each of the databases, differentiated by assessment method. While some databases did no show a monotonic increase with age, particularly when it concerned self-reported data for near vision difficulties (Fig. 2A and D), the estimated prevalence increased with age for all assessment methods and definitions. Confidence intervals around the predictions highlighted uncertainty, particularly at older ages and for self-reported near vision difficulties.

**Fig. 4:**
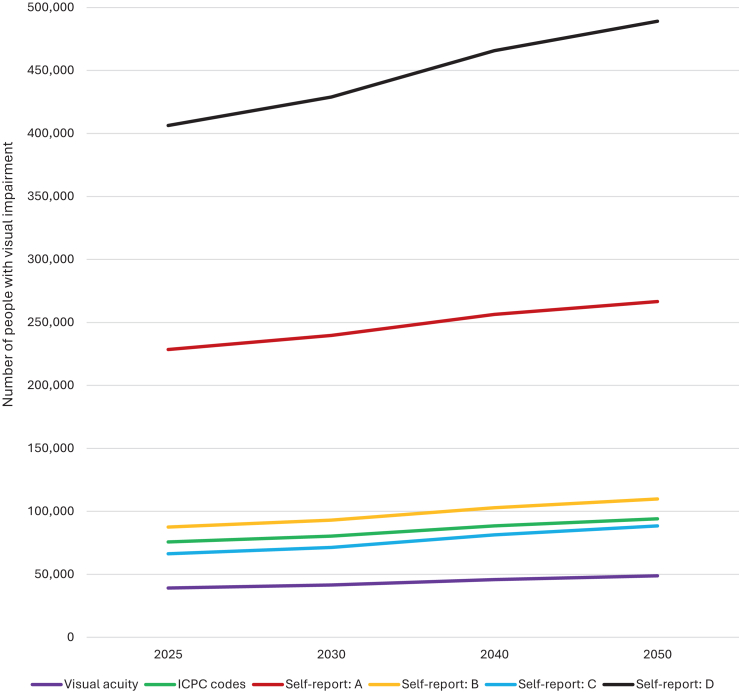
Number of people with visual impairment for different assessment methods^a^, from 2025 to 2050. ^a^Prevalence of visual impairment based on self-reported data is categorised by four definitions: A: only near vision difficulties; B: only distance vision difficulties; C: both near and distance vision difficulties; D: any vision difficulties. ICPC: International Classification of Primary Care.

Fig. 4 shows the number of cases with visual impairment stratified by assessment method, and projections to 2050; exact numbers can be found in Appendix 8. Depending on assessment method, 39,100–406,400 adults in the Netherlands have visual impairment in 2025. This number is projected to increase due to population growth and ageing, reaching 48,800–489,100 by 2050. Self-reported visual impairment, irrespective of the criteria, always resulted in higher numbers compared to visual acuity, although both near and distance vision difficulties (D) resulted in lower numbers than ICPC codes (respectively 66,300 and 75,700 currently and 88,400 and 94,100 by 2050). The largest increases were observed in older age groups, reflecting the growing proportion of older adults (Appendix 9).

## Discussion

This study provides current estimates of the prevalence of visual impairment in adults in the Netherlands and explores projections extending to 2050. The prevalence estimates range from 0.28–3.21%, depending on assessment method and definitions. We modelled the prevalence by age, showing an increase across all assessment methods and definitions as age increases. Our predictions indicate that 39,100–406,400 adults have visual impairment in 2025, and this number is expected to increase to 48,800–489,100 by 2050, depending on the assessment method and definition.

Prevalence of visual impairment varied by assessment method. Prevalence was lowest for databases with visual acuity data (0.28–0.30%), and highest for databases with self-reported data. For databases with visual acuity data, only best-corrected distance visual acuity was measured, not presenting visual acuity. Yet, self-reported visual functioning aligns more with presenting visual acuity, which might explain the discrepancy, as uncorrected refractive errors are a major cause of visual impairment, also in western Europe.^32^ Thus, best-corrected visual acuity may underestimate the true prevalence of visual impairment. We also noted differences in self-reported prevalence for the different definitions. Naturally, prevalence was highest when focussing on any vision difficulties (D, 3.21%), and lowest when both near and distance vision needed to be problematic (C, 0.51%). Here, the latter might best reflect best-corrected visual acuity, whereas the former might best reflect presenting visual acuity. The difference in prevalence between these definitions might suggest that uncorrected refractive errors may be an important cause of the self-reported difficulties.

Although our prevalence estimates vary for different definitions, they are in line with that of neighbouring countries. For example, the prevalence among working-age individuals in western Europe was 2.45% in 2019, based on best-corrected or presenting visual acuity.^33^ A population-based cohort study in Germany among adults aged 35–74 using best-corrected visual acuity estimated a prevalence of 0.37% between 2007–2012.^34^ From that same period, a study in 14 European population-based studies estimated a prevalence of 0.92% based on best-corrected visual acuity among adults aged 55+.^35^ The global burden of disease study suggests a prevalence of 3.78% for males aged 50+ and 4.37% for females aged 50+ in western Europe based on best-corrected or presenting visual acuity.^32^

Unsurprisingly, we found that prevalence increased with advancing age. Prevalence was 12% in the highest age category for the most liberal definition (i.e., self-reported data—any vision problems (D)). The prevalence for self-report was not always monotonically increasing with age, especially for near vision difficulties (A and D). Here, prevalence increased between ages 40–60, after which the prevalence decreased again. This trend can likely be attributed to the onset of presbyopia, where individuals may experience problems with near vision but do not have the correct correction yet, leading to an increase in self-reported vision problems. The decreasing prevalence after this age could reflect that appropriate refractive correction (e.g., reading glasses or contact lenses) is obtained, reducing perceived difficulties.

Depending on the assessment method and definition, we estimate that visual impairment affects 39,100–406,400 adults. Most estimates are lower than those forecasted in previous studies (i.e., 350,000–380,000 adults in 2020),^6^^,^^10^ despite an increased population growth. Potentially, new treatment options and higher quality ophthalmological care have resulted in less people with visual impairment.^14^ Other studies in western Europe also suggest declining trends.^33^^,^^35^ Alternatively, selection bias may have caused people with visual impairment to be underrepresented in the included databases (healthy-cohort effect), potentially resulting in underestimated prevalences. This does not hold for ICPC codes, which are complaints, symptoms and conditions registered by general practitioners. However, we only selected two codes most indicative for visual impairment. Other codes (e.g., F84-macular degeneration, F92-cataract, F93-glaucoma) might also suggest visual impairment but were excluded due to uncertainty about whether they consistently reflected impaired vision. Additionally, the accuracy of ICPC codes depends on the registration practices of general practitioners, which may also lead to underestimation.^36^

Population growth and demographic ageing cause a substantial increase in the number of people with visual impairment in the Netherlands, increasing 17–33% from 2025 to 2050, depending on assessment method and definition. Although limitations of projections need to be considered, they suggest a growing demand for eye care and low vision services in the coming decades. This rising burden may affect healthcare resources, impact quality of life, and increase societal costs. Investments in ophthalmological treatments and developing sustainable healthcare policies may be necessary to address these rising numbers and ensure long-term access to eye care and low vision services.

An important strength of our study is the large amount of data. We included data from 1,814,716 persons, which is 12% of the Dutch adult population. Although some overlap between databases existed, an empirical assessment of the three largest datasets (covering 97.9% of all individuals) showed that overlap was limited (3.3% overall and 0.8% among individuals with visual impairment), suggesting that any resulting bias is likely minimal. Moreover, because analyses were conducted at the study level, with each database contributing a single prevalence estimate, potential overlap does not result in double counting within the meta-analyses. Together with the partly distinct sampling approaches and the low overall prevalence of visual impairment, this supports the robustness of our pooled estimates. Thus, our extensive data offers potential for use in future studies that estimate the prevalence of visual impairment in Europe or globally. A second strength is that we applied external weighing to make data representative for the Dutch population for age and sex.

Limitations should also be noted. First, databases were identified primarily through expert consultation rather than a systematic literature search. We deliberately chose this approach because several key databases that include data on vision (e.g., GEMON and GECON) are not described in the scientific literature and would otherwise have been missed. Given that the Netherlands is a relatively small country with a limited number of institutions collecting population-based health data, we consider it unlikely that relevant databases were overlooked. Second, only two databases contained data on best-corrected visual acuity, and one database on ICPC codes, with accuracy of the latter being dependent on general practitioners' registration practices. The majority of the databases had self-reported data on visual functioning, which can be subject to considerably bias. Moreover, the content of questions and response options is open to interpretation and subjectivity as well, with terms as “small” and “difficult”. Nevertheless, studies have shown that self-reported vision using questionnaires often aligns with presenting visual acuity in population-based studies, especially in identifying normal vision.^37^, ^38^, ^39^ However, there is a tendency for over-identification of visual impairment. This might explain why prevalence rates based on self-reported data were higher than prevalence rates based on visual acuity or ICPC codes. On the other hand, the aforementioned selection bias might have resulted in underrepresentation of people with visual impairment, especially in longitudinal cohort studies with repeated measurements and extensive data collection procedures (i.e., The Maastricht Study, Rotterdam Study, Doetinchem Cohort Study, LASA, and Lifelines). Most of these databases have lower age-adjusted prevalence estimates than GEMON and GECON. The latter are cross-sectional surveys that can be completed online, through paper-and-pencil, or a telephone interview, and as such are less burdensome for people with visual impairment. It should be noted than none of the databases include institutionalised populations, where prevalences might be highest.^7^ Third, our study does not distinguish between underlying causes of visual impairment, although these are relevant from clinical and public health perspectives. Due to data limitations, analysis of these causes was not feasible, limiting insight into condition-specific prevalence or intervention targets. Regarding data quality, our access to the original databases was limited to de-identified data containing only the variables required for our analyses. As a result, we were not able to evaluate completeness or internal validation procedures at the database level. However, all included datasets are maintained by reputable national health authorities or academic institutions, and we relied on the data custodians’ established quality assurance procedures. Fourth, there was a large amount of heterogeneity between databases. Assessment method partly explained heterogeneity, but residual heterogeneity remained, caused by differences in study design and sampling. Additionally, there are also potential differences between databases in terms of health status of participants, possibly impacting visual impairment prevalence. Besides age and sex, data on other characteristics were not uniformly available for each database, and therefore could not be included or corrected for in our study. Fifth, we only meta-analysed the prevalence of visual impairment (including blindness), and not of blindness separately because of the limited amount of data. We acknowledge that visual acuity is variable by nature, though we do not consider that this materially affects our conclusions, since the thresholds for visual impairment and blindness are beyond typical measurement variation.^40^ Sixth, our prevalence estimates vary depending on the definition that we used, resulting in absolute values that represent a broad range, reflecting substantial uncertainty in the number of people estimated to have visual impairment. Moreover, confidence intervals for these numbers are wide as well, underscoring the uncertainty of the projected numbers. Finally, the forecast of visual impairment must be interpreted with caution. While we accounted for changing age distributions and population growth projections, these have previously proven to be underestimations.^6^^,^^10^ Moreover, we could not model future changes in other relevant population characteristics, such as ethnicity, health status, and myopia prevalence.^41^ Although we initially considered including earlier time points to assess trends, we found no consistent evidence of temporal change in visual impairment prevalence after adjusting for age and sex. Therefore, we applied demographic projections only. Whether age-adjusted prevalence is increasing or decreasing over time remains unclear and may depend on such as better treatment options and higher life-expectancy. Moreover, we did not model future developments in treatment or ophthalmological care. As such, our projections assume stable conditions and do not account for changes in clinical practice or treatment policies.

In conclusion, this study provides comprehensive and up-to-date estimates of the prevalence of visual impairment in adults in the Netherlands and explores how this burden may evolve due to population growth and ageing. While prevalence estimates varied depending on assessment method, our findings align with those reported in neighbouring countries. Despite inherent limitations, our study offers insights into the potential future burden of visual impairment by providing trends and plausible ranges. As visual impairment impacts quality of life, these findings support scenario-based planning and highlight the importance of continued attention to prevention, as well as access to eye care and low vision services at national level.

## Contributors

RvN, CK, JK, RW, TBr, CvdW, MM, and PvE conceptualised the study; RvN, HvdA, and TRK acquired the funding; EE curated and visualised the data; EE and JH conducted the formal analyses, were responsible for the methodology and the software; EE, JV, HP, WV, VdV, WR, TBe, and MH conducted the investigation; EE and TRK were responsible for the project administration; EE, JV, HP, WV, VdV, WR, TBe, MH, and RvN provided the resources for this study; RvN provided supervision; JH, RvN, JV, HP, WV, VdV, WR, TBe, and MH validated the data; EE wrote the original draft of the manuscript; all authors reviewed and edited the manuscript. EE, JV, HP, WV, VdV, WR, TBe, MH, and RvN accessed and verified the underlying data. The authors had full access to the data in the study. EE and RvN had the final responsibility for the decision to submit for publication.

## Data sharing statement

Individual participant data and a data dictionary defining each field will not be made available to others. The study protocol, statistical analysis plan, and analytic code will be made available to others with publication. This data will be made available after requests have been made to Ellen Elsman via email (e.elsman@amsterdamumc.nl) at no additional restrictions.

## Declaration of interests

RPL Wisse receives consulting fees from Alcon BV, Carl Zeiss GmbH, and Easee BV. RMA van Nispen receives consulting fees from Janssen-Cilag NV. CCW Klaver and V de Vries declare that the Rotterdam Study is supported by Oogfonds, Stichting voor Ooglijders, Stichting voor Blindenhulp, Stichting Lijf en Leven, Henkes stichting, and Landelijke Stichting voor Blinden en Slechtzienden (LSBS). Additional support was given by Erasmus Medical Center, Erasmus University, Netherlands Organisation for the Health Research and Development (ZonMw), the Research Institute for Diseases in the Elderly (RIDE), the Ministry of Education, Culture and Science, the Ministry for Health, Welfare and Sports, the European Commission (DG XII), and the Municipality of Rotterdam. MB Muijzer reports a patent (pending): P096387NL, not related to the study. No specific funding was received for this particular project. EBM Elsman, TP Rausch-Koster, HPA van der Aa, J Hoogland, HSJ Picavet, WMM Verschuren, TTJM Berendschot, JW Vanhommerig, WD Ramdas, M Huisman, T Brussee, C van der Windt, P van Es, and JEE Keunen declare no competing interests.

## Appendix A Supplementary data

Supplementary Material

Translated Abstract
