# Supplementary material for: Estimating the prevalence of visual impairment in the Netherlands, with forecasts up to 2050: a meta-analysis of national databases
Source: eClinicalMedicine. 2026 Apr 1;94:103858. doi: 10.1016/j.eclinm.2026.103858 (PMC13087707; doi:10.1016/j.eclinm.2026.103858)
Supplement: Supplementary Material [file mmc1.pdf]

## Supplemental materials

**Table of contents**


| | | |
|---|---|---|
| **Appendix 1** | **Description of included databases, ethical approval, and other information** | **p. 2** |
| **Appendix 2** | **Information on sampling frame, recruitment, and representativeness of included databases** | **p. 5** |
| **Appendix 3** | **References to statistical methods, software, and packages used** | **p. 6** |
| **Appendix 4** | **Descriptive characteristics (unweighted) of included databases** | **p. 7** |
| **Appendix 5** | **Stratified prevalence of visual impairment by database** | **p. 11** |
| **Appendix 6** | **Pooled prevalence estimates of visual impairment from meta-analyses differing in self-report definition, assessment method, and transformation** | **p. 12** |
| **Appendix 7** | **Sensitivity analysis: meta-analysis restricted to self-report studies for definition C** | **p. 13** |
| **Appendix 8** | **Estimated number of adults with visual impairment in the Netherlands (2025-2050)** | **p. 14** |
| **Appendix 9** | **Stratified projected number of adults with visual impairment by age group and assessment method (2025-2050)** | **p. 15** |

## Appendix 1. Description of included databases, ethical approval, and other information

| Database | Description | Ethical approval | Other information |
|---|---|---|---|
| The Maastricht Study[1] | The Maastricht Study is an observational prospective population-based cohort study. The study focuses on the aetiology, pathophysiology, complications and comorbidities of type 2 diabetes mellitus (T2DM) and is characterised by an extensive phenotyping approach. Eligible for participation were all individuals aged between 40 and 75 years and living in the southern part of the Netherlands. Participants were recruited through mass media campaigns and from the municipal registries and the regional Diabetes Patient Registry via mailings. Recruitment was stratified according to known T2DM status, with an oversampling of individuals with T2DM, for reasons of efficiency. | The study has been approved by the institutional medical ethical committee (NL31329.068.10) and the Minister of Health, Welfare and Sports of the Netherlands (Permit 131088-105234-PG). All participants gave written informed consent. | The study was supported by the European Regional Development Fund via OP-Zuid, the Province of Limburg, the Dutch Ministry of Economic Affairs (grant 31O.041), Stichting De Weijerhorst (Maastricht, The Netherlands), the Pearl String Initiative Diabetes (Amsterdam, The Netherlands), the Cardiovascular Centre (CVC, Maastricht, the Netherlands), CARIM School for Cardiovascular Diseases (Maastricht, The Netherlands), CAPHRI Care and Public Health Research Institute (Maastricht, The Netherlands), NUTRIM School for Nutrition and Translational Research in Metabolism (Maastricht, the Netherlands), Stichting Annadal (Maastricht, The Netherlands), Health Foundation Limburg (Maastricht, The Netherlands), and by unrestricted grants from Janssen-Cilag B.V. (Tilburg, The Netherlands), Novo Nordisk Farma B.V. (Alphen aan den Rijn, the Netherlands), and Sanofi-Aventis Netherlands B.V. (Gouda, the Netherlands). |
| Rotterdam Study[2] | The Rotterdam Study is a prospective cohort study ongoing since 1990 in the city of Rotterdam in The Netherlands. The study targets cardiovascular, endocrine, hepatic, neurological, ophthalmic, psychiatric, dermatological, otolaryngological, locomotor, and respiratory diseases. | The Rotterdam Study has been approved by the institutional review board (Medical Ethics Committee, registration number MEC 02.1015) of the Erasmus Medical Centre and by the review board of The Netherlands Ministry of Health, Welfare and Sports (license number 1071272-159521-PG). The RS is entered into the Dutch National Trial Register (NTR; www.onderzoekmetmensen.nl) and the WHO International Clinical Trials Registry Platform (ICTRP; www.who.int/clinical-trials-registry-plotform) under shared catalogue number NTR6831. | |
| NPCD (Nivel Primary Care Database)[3,4] | The Nivel Primary Care Database uses routinely recorded data from health care providers to monitor health and utilisation of health services in a representative sample of the Dutch population. This includes data from general practitioners. The network design takes account of the geographical distribution of the population and its distribution over areas with different degrees of population density. | This study has been approved according to the governance code of Nivel Primary Care Database, under number NZR-00323.039. | The use of electronic health records for research purposes is allowed under certain conditions. When these conditions are fulfilled, neither obtaining informed consent from patients nor approval by a medical ethics committee is obligatory for this type of observational studies containing no directly identifiable data (art. 24 GDPR Implementation Act jo art. 9.2 sub j GDPR). |
| GEMON (Dutch Public Health monitor)[5] | The Dutch Public Health Monitor started in 2012. This is an extensive data collection consisting of the quadrennial questionnaires of the Municipal Health Services (GGD) and the annual national health questionnaire of Statistics Netherlands (CBS). The resulting database contains figures on lifestyle and other health-related aspects at national, regional, and municipal level. | The Public Health Monitor 2012, 2016 and 2020 is reviewed by the ethical committee of the Amsterdam Medical Center, which declared it non-WMO obligated. This means it is exempt from further ethical review because it is not subject to the Dutch Medical Research with Human Subjects Law. Those invited to partake in the study received a letter outlining study's purpose, emphasizing the voluntary nature of participation without the need for providing a reason for refusal. | |

| | | | |
|---|---|---|---|
| | | Efforts have been made to minimize the potential identifiability of individuals, and researchers are provided with an anonymized dataset. Stringent confidentiality measures bind researchers, and a data protection impact assessment was conducted. In addition, research findings are only reported at the group level, ensuring that individuals remain unidentifiable. | |
| GECON (Dutch Health Survey)[6] | The Dutch Health Survey is a continuous sample survey conducted by Statistics Netherlands (CBS). The aim of the health survey is to describe the health, lifestyle and use of healthcare by the Dutch population. Because the health survey is performed repeatedly, time series are created that relate to the development of the variables describe health, lifestyle and healthcare use over time as accurately as possible. | The Dutch Health Survey (GECON) is conducted under the legal framework of the Dutch Public Health Act (*Wet publieke gezondheid*). As the data collection is carried out on this statutory basis, approval from a Medical Research Ethics Committee was not required. | |
| Doetinchem Cohort Study[7,8] | The Doetinchem Cohort Study is a population-based prospective study that studies the impact of (changes in) lifestyle factors and biological risk factors on various aspects of health and well-being of Dutch adults living in Doetinchem. It currently consists of six repeated measures with 5-year intervals over a 30-year period. In 1987–1991, self-completed questionnaires were collected, and a physical examination was performed on a random sample of 12,404 (response rate: 62%) participants aged 20–59 years from the town of Doetinchem. A random sample was re-invited for examination every 5 years. | The Doetinchem Cohort Study was performed in accordance with the Declaration of Helsinki and approval was obtained from the external Medical Ethics Committee of The Netherlands Organisation for Applied Scientific Research and the University of Utrecht. Informed consent was obtained from all individual participants included in the study. | |
| LASA (Longitudinal Aging Study Amsterdam)[9,10] | The LASA Study is a prospective cohort study of older adults in the Netherlands, initially based on a nationally representative sample of 3805 people aged between 55 and 85 years. The study has been ongoing since 1992, and every 10 years a new cohort is added (n=1002 for cohort 2 and n=1023 for cohort 3). The study focuses on the determinants, trajectories and consequences of physical, cognitive, emotional and social functioning. | The LASA study is conducted in line with the Declaration of Helsinki and received approval by the medical ethics committee of the VU University Medical Centre (IRB numbers: 92/138, 2002/141, 2012/361, and 2016.301) | The Longitudinal Aging Study Amsterdam is supported by grants from the Netherlands Ministry of Health, Welfare and Sport, Directorate of Long-Term Care. |
| Lifelines[11,12] | Lifelines is a multi-disciplinary prospective population-based cohort study examining in a unique three-generation design the health and health-related behaviours of 167,729 persons living in the North of the Netherlands. It employs a broad range of investigative procedures in assessing the biomedical, socio-demographic, behavioural, physical and psychological factors which contribute to the health and disease of the general population, with a special focus on multi-morbidity and complex genetics. | The Lifelines protocol has been approved by the UMCG Medical ethical committee under number 2007/152. | The Lifelines initiative has been made possible by subsidy from the Dutch Ministry of Health, Welfare and Sport, the Dutch Ministry of Economic Affairs, the University Medical Centre Groningen (UMCG), Groningen University and the Provinces in the North of the Netherlands (Drenthe, Friesland, Groningen). |

## References

1. Schram MT, Sep SJ, Van Der Kallen CJ, et al. The Maastricht Study: an extensive phenotyping study on determinants of type 2 diabetes, its complications and its comorbidities. *European journal of epidemiology* 2014;**29**:439-51.
2. Ikram MA, Kieboom BC, Brouwer WP, et al. The Rotterdam Study. Design update and major findings between 2020 and 2024. *European Journal of Epidemiology* 2024;**39**(2):183-206.
3. Nivel. Nivel Zorgregistraties. 2020, 2021, 2022;**NZR-003.23.0.39**.
4. Schweikardt C, Verheij RA, Donker GA, Coppieters Y. The historical development of the Dutch Sentinel General Practice Network from a paper-based into a digital primary care monitoring system. *Journal of Public Health* 2016;**24**:545-62.
5. Public Health Monitor 2020 of the Community Health Services, Statistics Netherlands and the National Institute for Public Health and the Environment.
6. van den Brakel J. Aanvullende analyses variabelen omtrent bewegen uit de Gezondheidsenquête.
7. Verschuren W, Blokstra A, Picavet H, Smit H. Cohort profile: the Doetinchem cohort study. *International journal of epidemiology* 2008;**37**(6):1236-41.
8. Picavet HSJ, Blokstra A, Spijkerman AM, Verschuren WM. Cohort profile update: the Doetinchem Cohort Study 1987–2017: lifestyle, health and chronic diseases in a life course and ageing perspective. *International journal of epidemiology* 2017;**46**(6):1751-g.
9. Huisman M, Poppelaars J, van der Horst M, et al. Cohort profile: the longitudinal aging study Amsterdam. *International journal of epidemiology* 2011;**40**(4):868-76.
10. Hoogendijk EO, Deeg DJ, de Breij S, et al. The Longitudinal Aging Study Amsterdam: cohort update 2019 and additional data collections. *European journal of epidemiology* 2020;**35**(1):61-74.
11. Scholtens S, Smidt N, Swertz MA, et al. Cohort Profile: LifeLines, a three-generation cohort study and biobank. *International journal of epidemiology* 2015;**44**(4):1172-80.
12. Sijtsma A, Rienks J, van der Harst P, Navis G, Rosmalen JG, Dotinga A. Cohort Profile Update: Lifelines, a three-generation cohort study and biobank. *International journal of epidemiology* 2022;**51**(5):e295-e302.

## Appendix 2. Information on sampling frame, recruitment, and representativeness of included databases

| Database | Target population / sampling frame | Geographical coverage | Recruitment | Sample size included in current study | Response / retention | Representativeness – strengths | Representativeness – limitations |
|---|---|---|---|---|---|---|---|
| The Maastricht Study | Adults 40-79 years | South of the Netherlands (regional) | Municipality registries; media campaign; oversampling of T2DM | 7,739 | Ongoing cohort follow-up | Representative for South Limburg region (age, sex, ethnicity, urbanisation) | Regional; age-restricted; oversampling of T2DM |
| Rotterdam Study | Adults ≥40 years | Ommoord district (Rotterdam) | Municipality registry; multiple cohort extensions | 7,687 | Overall ~65%; | Well-defined population-based cohort; high follow-up | Regional; age-restricted |
| NPCD (Nivel Primary Care Database) | GP patients from registered practices | Nationwide | GP practices | 1,240,330 | Continuous routine registration | Broad national coverage (~10% of Dutch population); representative for age, sex, urbanisation | |
| GEMON (Dutch Public Health Monitor) | Adults ≥18 years | Nationwide | Random sample from population register; mixed-mode survey (online, paper, home visit) | 522,015 | ~38–40% | Designed for national and regional representativeness; large sample | Moderate response rate; excludes institutionalised populations |
| GECON (Dutch Health Survey) | Adults ≥18 years in private households | Nationwide | Random sample from population register; mixed-mode survey (online, paper, home visit) | 21,917 (3 years pooled) | <50% | National probability sample; suitable for national prevalence estimates | Moderate response rate; excludes institutionalised populations |
| Doetinchem Cohort Study | Originally 20–59 years at baseline (1987); currently aging cohort | Doetinchem (Eastern NL) | Municipality registry; stratified by age/sex | 2,748 | Baseline 62%; follow-up 75-80% | Long-term follow-up; stratified sampling | Regional, single-town cohort; aging cohort |
| LASA | Originally 55-84 years; aging cohort | Amsterdam and surrounding area | Municipality registry; multiple cohort extensions | 1,047 | Baseline ~60%; follow-up 60–70% | Originally nationally stratified sample of older adults | Age-restricted |
| Lifelines | Adults ≥18 years (three-generation design) | North of the Netherlands | GP recruitment, family inclusion, self-registration | 18,301 | Baseline ~70%; follow-up 58–84% | Representative for Northern NL | Regional |

**Appendix 3. References to statistical methods, software, and packages used**

| Statistical method | Reference |
|---|---|
| External weighing | LASA. Weight factors 2019 [cited 2025 25 February]. Available from: https://lasa-vu.nl/topics/weight-factors/. |
| Wilson method | Wilson EB. Probable inference, the law of succession, and statistical inference. Journal of the American Statistical Association 1927;22(158):209-12. |
| Logit transformation | Lipsey MW, Wilson DB. Practical meta-analysis. 2001, Sage Publications. |
| DerSimoninan and Laird method | DerSimonian R, Laird N. Meta-analysis in clinical trials. Controlled clinical trials 1986;7(3):177-88. |
| **Statistical software** | **Reference** |
| SPSS version 28 | IBM Corp. Released 2021. IBM SPSS Statistics for Windows, Version 28.0. Armonk, NY: IBM Corp. |
| R version 4.0.5 | R Core Team. (2021). R: A language and environment for statistical computing. R Foundation for Statistical Computing, Vienna, Austria. |
| **Package** | **Reference** |
| meta version 8.1-0 | Balduzzi S, Rücker G, Schwarzer G. How to perform a meta-analysis with R: a practical tutorial. BMJ Ment Health 2019;22(4):153-60. |
| metafor version 4.8-0 | Viechtbauer W. Conducting meta-analyses in R with the metafor package. Journal of statistical software 2010;36:1-48. |
| lme4 version 1.1-26 | Bates D, Mächler M, Bolker B, Walker S. Fitting linear mixed-effects models using lme4. Journal of statistical software 2015;67:1-48. |
| ggeffects version 2.0.0 | Lüdecke D. ggeffects: Tidy data frames of marginal effects from regression models. Journal of open source software 2018;3(26):772. |
| ggplot2 version 3.3.3 | Villanueva RAM, Chen ZJ. ggplot2: elegant graphics for data analysis. Taylor & Francis; 2019. |

**Appendix 4. Descriptive characteristics (unweighted) of included databases**

| Database | Database type | Period last data collection | Type of vision data | Characteristics of study population | | |
|---|---|---|---|---|---|---|
| | | | | n | % Female | Age, mean (SD) [range] |
| The Maastricht Study | Prospective population-based cohort study | 2011-2020 | Best corrected visual acuity: ETDRS chart | 7,739 | 50·7 | 59·5 (8·8) [40-79] |
| Rotterdam Study | Prospective population-based cohort study | 2010-2020 | Best corrected visual acuity: ETDRS chart | 7,687 | 56·7 | 64·5 (11·8) [41-100] |
| NPCD (Nivel Primary Care Database) | Registration data general practices | 2022 | ICPC codes | 1,240,330 | 51·0 | 18-25: 129,267 (10·4)[a]<br>25-30: 94,785 (7·6)<br>30-35: 99,266 (8·0)<br>35-40: 93,729 (7·6)<br>40-45: 90,352 (7·3)<br>45-50: 90,833 (7·3)<br>50-55: 110,339 (8·9)<br>55-60: 110,249 (8·9)<br>60-65: 103,323 (8·3)<br>65-70: 90,634 (7·3)<br>70-75: 81,812 (6·6)<br>75-80: 67,710 (5·5)<br>80-85: 41,935 (3·4)<br>85+: 36,096 (2·9) |
| GEMON (Dutch Public Health Monitor) | Cross-sectional health survey | 2020 | Self-report: 2 questions | 522,015 | 53·1 | 58·5 (18·4) [29-79][b] |
| GECON (Dutch Health Survey) | Cross-sectional health survey | 2020-2022 | Self-report: 2 questions | 21,917 | 51·0 | 51·9 (18·3) [26-75][b] |
| Doetinchem Cohort Study | Prospective population-based cohort study | 2018-2022 | Self-report: 2 questions | 2748 | 52·5 | 67·7 (8·5) [51-90] |
| LASA (Longitudinal Aging Study Amsterdam) | Prospective population-based cohort study | 2021-2022 | Self-report: 2 questions | 1,047 | 51·0 | 75·1 (7·0) [63-97] |
| Lifelines | Prospective population-based cohort study | 2021-2024 | Self-report: NEI VFQ-25[c] | 18,301 | 59·9 | 59·4 (11·3) [20-96] |

[a] Data represented as n (%)

[b] Range represents the $10^{th}$-$90^{th}$ percentile

[c] Not all items from the NEI VFQ-25 were administered, see eye_conditions_oq [Lifelines Wiki] (rug.nl)

ETDRS: Early Treatment Diabetic Retinopathy Study; ICPC: International Classification of Primary Care; NEI VFQ-25: National Eye Institute 25-item Vision Function Questionnaire

**Appendix 5. Stratified prevalence of visual impairment by database**

Prevalence estimates (age-and sex-standardised) and 95% confidence intervals are shown by sex, age group, and working age for each database. Visual impairment is defined according to the assessment method used in each database (self-report, visual acuity, or ICPC codes).

| | The Maastricht Study (n=7,739) | | | | Rotterdam Study (n=7,684) | | | | NPCD (Nivel Primary Care Database) (n=1,240,330) |
|---|---|---|---|---|---|---|---|---|---|
| | **Mild visual impairment (visual acuity)** | **Moderate visual impairment (visual acuity)** | **Severe visual impairment (visual acuity)** | **Blindness (visual acuity)** | **Mild visual impairment (visual acuity)** | **Moderate visual impairment (visual acuity)** | **Severe visual impairment (visual acuity)** | **Blindness (visual acuity)** | **Visual impairment (ICPC codes)** |
| **Strata** | **Prevalence, % (95% CI)** | **Prevalence, % (95% CI)** | **Prevalence, % (95% CI)** | **Prevalence, % (95% CI)** | **Prevalence, % (95% CI)** | **Prevalence, % (95% CI)** | **Prevalence, % (95% CI)** | **Prevalence, % (95% CI)** | **Prevalence, % (95% CI)** |
| **Total** | 0·7 (0·5-0·9) | 0·2 (0·1-0·3) | 0·0 (0·0-0·0) | 0·0 (0·0-0·0) | 0·4 (0·3-0·6) | 0·4 (0·3-0·6) | 0·0 (0·0-0·0) | 0·1 (0·1-0·2) | 0·5 (0·5-0·5) |
| **Sex** | | | | | | | | | |
| **Male** | 0·9 (0·6-1·3) | 0·2 (0·1-0·4) | 0·0 (0·0-0·1) | 0·0 (0·0-0·1) | 0·3 (0·2-0·5) | 0·5 (0·3-0·8) | 0·0 (0·0-0·1) | 0·0 (0·0-0·1) | 0·5 (0·5-0·5) |
| **Female** | 0·5 (0·3-0·8) | 0·1 (0·0-0·3) | 0·0 (0·0-0·1) | 0·0 (0·0-0·1) | 0·4 (0·2-0·6) | 0·3 (0·2-0·5) | 0·0 (0·0-0·1) | 0·1 (0·0-0·3) | 0·5 (0·5-0·5) |
| **Age (in years)** | | | | | | | | | |
| **18-24** | | | | | | | | | 0·4 (0·4-0·4) |
| **25-29** | | | | | | | | | 0·2 (0·2-0·2) |
| **30-34** | | | | | | | | | 0·2 (0·2-0·2) |
| **35-39** | | | | | | | | | 0·2 (0·2-0·2) |
| **40-44** | 0·0 (0·0-0·3) | 0·5 (0·2-1·1) | 0·0 (0·0-0·3) | 0·0 (0·0-0·3) | 0·0 (0·0-0·8) | 0·0 (0·0-0·8) | 0·0 (0·0-0·8) | 0·0 (0·0-0·8) | 0·2 (0·2-0·2) |
| **45-49** | 0·6 (0·3-1·2) | 0·0 (0·0-0·3) | 0·0 (0·0-0·3) | 0·0 (0·0-0·3) | 0·1 (0·0-0·8) | 0·1 (0·0-0·8) | 0·0 (0·0-0·6) | 0·0 (0·0-0·6) | 0·3 (0·3-0·3) |
| **50-54** | 0·5 (0·2-1·1) | 0·1 (0·0-0·5) | 0·0 (0·0-0·3) | 0·0 (0·0-0·3) | 0·0 (0·0-0·3) | 0·0 (0·0-0·3) | 0·0 (0·0-0·3) | 0·0 (0·0-0·3) | 0·4 (0·4-0·4) |
| **55-59** | 0·3 (0·1-0·8) | 0·0 (0·0-0·3) | 0·0 (0·0-0·3) | 0·0 (0·0-0·3) | 0·1 (0·0-0·5) | 0·2 (0·1-0·7) | 0·1 (0·0-0·5) | 0·0 (0·0-0·3) | 0·4 (0·4-0·4) |
| **60-64** | 0·3 (0·1-0·9) | 0·3 (0·1-0·9) | 0·0 (0·0-0·4) | 0·0 (0·0-0·4) | 0·1 (0·0-0·5) | 1·3 (0·8-2·2) | 0·0 (0·0-0·4) | 0·2 (0·1-0·7) | 0·5 (0·5-0·5) |
| **65-69** | 0·9 (0·5-1·7) | 0·3 (0·1-0·9) | 0·0 (0·0-0·4) | 0·0 (0·0-0·4) | 0·3 (0·1-0·9) | 0·2 (0·1-0·7) | 0·0 (0·0-0·4) | 0·1 (0·0-0·6) | 0·7 (0·6-0·8) |
| **70-74** | 1·3 (0·7-2·4) | 0·2 (0·0-0·9) | 0·0 (0·0-0·5) | 0·0 (0·0-0·5) | 0·3 (0·1-1·0) | 0·0 (0·0-0·5) | 0·0 (0·0-0·5) | 0·0 (0·0-0·5) | 0·9 (0·8-1·0) |
| **75-79** | 4·5 (2·7-7·5) | 0·0 (0·0-1·3) | 0·0 (0·0-1·3) | 0·0 (0·0-1·3) | 0·5 (0·2-1·5) | 0·4 (0·1-1·4) | 0·0 (0·0-0·7) | 0·1 (0·0-0·9) | 1·1 (1·0-1·2) |
| **80-84** | | | | | 1·7 (0·8-3·5) | 0·6 (0·2-2·0) | 0·1 (0·0-1·1) | 0·1 (0·0-1·1) | 1·5 (1·4-1·6) |
| **85-89** | | | | | 3·1 (1·5-6·2) | 2·2 (1·0-5·0) | 0·0 (0·0-1·6) | 0·2 (0·0-2·0) | 1·9 (1·8-2·0)[c] |
| **90-94** | | | | | 1·9 (0·4-7·9)[b] | 1·9 (0·4-7·9)[b] | 0·0 (0·0-4·7)[b] | 0·0 (0·0-4·7)[b] | |
| **95+** | | | | | | | | | |
| **Working age** | | | | | | | | | |
| **Yes** | 0·5 (0·3-0·7) | 0·2 (0·1-0·4) | 0·0 (0·0-0·1) | 0·0 (0·0-0·1) | 0·1 (0·0-0·2) | 0·0 (0·0-0·1) | 0·3 (0·2-0·5) | 0·1 (0·0-0·2) | 0·3 (0·3-0·3) |
| **No** | 1·0 (0·7-1·5) | 0·1 (0·0-0·3) | 0·0 (0·0-0·2) | 0·0 (0·0-0·2) | 0·8 (0·5-1·2) | 0·0 (0·0-0·1) | 0·5 (0·3-0·8) | 0·1 (0·0-0·3) | 1·1 (1·1-1·1) |

## Appendix 5. Continued

| | GEMON (Dutch Public Health Monitor)[a] (n=514,967) | | | | GECON (Dutch Health Survey)[a] (n=21,917) | | | |
|---|---|---|---|---|---|---|---|---|
| | Visual impairment (self-report: A) | Visual impairment (self-report: B) | Visual impairment (self-report: C) | Visual impairment (self-report: D) | Visual impairment (self-report: A) | Visual impairment (self-report: B) | Visual impairment (self-report: C) | Visual impairment (self-report: D) |
| **Strata** | **Prevalence, % (95% CI)** | **Prevalence, % (95% CI)** | **Prevalence, % (95% CI)** | **Prevalence, % (95% CI)** | **Prevalence, % (95% CI)** | **Prevalence, % (95% CI)** | **Prevalence, % (95% CI)** | **Prevalence, % (95% CI)** |
| **Total** | 2·8 (2·8-2·8) | 0·8 (0·8-0·8) | 1·0 (1·0-1·0) | 4·6 (4·5-4·7) | 1·9 (1·7-2·1) | 0·6 (0·5-0·7) | 0·7 (0·6-0·8) | 3·2 (3·0-3·4) |
| **Sex** | | | | | | | | |
| **Male** | 2·9 (2·8-3·0) | 0·4 (0·4-0·4) | 0·7 (0·7-0·7) | 4·0 (3·9-4·1) | 1·9 (1·7-2·2) | 0·3 (0·2-0·4) | 0·5 (0·4-0·7) | 2·7 (2·4-3·0) |
| **Female** | 2·8 (2·7-2·9) | 1·2 (1·2-1·2) | 1·3 (1·3-1·3) | 5·2 (5·1-5·3) | 2·0 (1·8-2·3) | 0·8 (0·7-1·0) | 0·9 (0·7-1·1) | 3·7 (3·4-4·1) |
| **Age (in years)** | | | | | | | | |
| **18-24** | 0·6 (0·5-0·7) | 1·1 (1·0-1·2) | 0·5 (0·4-0·6) | 2·2 (2·1-2·3) | 0·4 (0·2-0·7)[d] | 0·7 (0·5-1·0)[d] | 0·3 (0·2-0·5)[d] | 1·3 (1·0-1·7)[d] |
| **25-29** | 0·3 (0·3-0·4) | 0·8 (0·7-0·9) | 0·5 (0·4-0·6) | 1·6 (1·5-1·7) | | | | |
| **30-34** | 0·4 (0·3-0·5) | 0·8 (0·7-0·9) | 0·5 (0·4-0·6) | 1·7 (1·6-1·8) | 0·2 (0·1-0·4) | 0·5 (0·3-0·8) | 0·3 (0·2-0·5) | 1·0 (0·7-1·4) |
| **35-39** | 0·6 (0·5-0·7) | 0·6 (0·5-0·7) | 0·5 (0·4-0·6) | 1·8 (1·7-1·9) | | | | |
| **40-44** | 1·4 (1·3-1·5) | 0·6 (0·5-0·7) | 0·6 (0·5-0·7) | 2·7 (2·5-2·9) | 1·7 (1·3-2·2) | 0·4 (0·2-0·7) | 0·4 (0·2-0·7) | 2·6 (2·1-3·2) |
| **45-49** | 4·1 (3·9-4·3) | 0·7 (0·6-0·8) | 0·7 (0·6-0·8) | 5·5 (5·3-5·7) | | | | |
| **50-54** | 5·3 (5·1-5·5) | 0·6 (0·5-0·7) | 0·8 (0·7-0·9) | 6·8 (6·6-7·0) | 3·1 (2·6-3·7) | 0·5 (0·3-0·8) | 0·6 (0·4-0·9) | 4·2 (3·6-4·9) |
| **55-59** | 4·5 (4·3-4·7) | 0·6 (0·5-0·7) | 0·9 (0·8-1·0) | 5·9 (5·7-6·1) | | | | |
| **60-64** | 4·0 (3·8-4·2) | 0·6 (0·5-0·7) | 1·0 (0·9-1·1) | 5·6 (5·4-5·8) | 2·3 (1·8-2·9) | 0·4 (0·2-0·7) | 0·6 (0·4-0·9) | 3·4 (2·8-4·1) |
| **65-69** | 3·2 (3·0-3·4) | 0·6 (0·5-0·7) | 0·9 (0·8-1·0) | 4·7 (4·5-4·9) | | | | |
| **70-74** | 3·0 (2·8-3·2) | 0·8 (0·7-0·9) | 1·1 (1·0-1·2) | 4·9 (4·7-5·1) | 2·6 (2·1-3·3) | 0·7 (0·4-1·1) | 0·8 (0·5-1·2) | 4·1 (3·4-4·9) |
| **75-79** | 3·3 (3·1-3·5) | 0·9 (0·8-1·0) | 1·6 (1·4-1·8) | 5·8 (5·5-6·1) | | | | |
| **80-84** | 4·6 (4·3-4·9) | 1·4 (1·2-1·6) | 2·7 (2·5-3·0) | 8·7 (8·3-9·1) | 5·0 (3·9-6·4) | 1·0 (0·6-1·8) | 2·9 (2·1-4·1) | 9·0 (7·5-10·8) |
| **85-89** | 6·6 (6·1-7·1) | 2·2 (1·9-2·5) | 4·9 (4·5-5·3) | 13·7 (13·0-14·4) | | | | |
| **90-94** | 9·5 (8·6-10·5) | 3·1 (2·6-3·7) | 8·3 (7·5-9·2) | 20·9 (19·7-22·2) | 8·0 (5·0-12·5)[b] | 1·9 (0·7-4·8)[b] | 10·7 (7·2-15·6)[b] | 20·5 (15·6-26·5)[b] |
| **95+** | 12·8 (10·9-15·0) | 2·6 (1·8-3·8) | 15·8 (13·7-18·2) | 31·1 (28·3-34·0) | | | | |
| **Working age** | | | | | | | | |
| **Yes** | 2·5 (2·5-2·5) | 0·7 (0·7-0·7) | 0·7 (0·7-0·7) | 3·9 (3·8-4·0) | 1·6 (1·4-1·8) | 0·5 (0·4-0·6) | 0·5 (0·4-0·6) | 2·5 (2·3-2·8) |
| **No** | 3·9 (3·8-4·0) | 1·1 (1·0-1·2) | 2·0 (1·9-2·1) | 7·0 (6·9-7·1) | 3·2 (2·8-3·7) | 0·7 (0·5-1·0) | 1·6 (1·3-2·0) | 5·4 (4·8-6·0) |

## Appendix 5. Continued

| Strata | Doetinchem Cohort Study[a] (n=2,731) | | | | LASA (Longitudinal Aging Study Amsterdam)[a] (n=1,047) | | | |
|---|---|---|---|---|---|---|---|---|
| | Visual impairment (self-report: A) | Visual impairment (self-report: B) | Visual impairment (self-report: C) | Visual impairment (self-report: D) | Visual impairment (self-report: A) | Visual impairment (self-report: B) | Visual impairment (self-report: C) | Visual impairment (self-report: D) |
| | Prevalence, % (95% CI) | Prevalence, % (95% CI) | Prevalence, % (95% CI) | Prevalence, % (95% CI) | Prevalence, % (95% CI) | Prevalence, % (95% CI) | Prevalence, % (95% CI) | Prevalence, % (95% CI) |
| **Total** | 5·3 (4·5-6·2) | 0·6 (0·4-1·0) | 0·5 (0·3-0·8) | 6·5 (5·6-7·5) | 0·5 (0·2-1·1) | 1·0 (0·6-1·8) | 0·6 (0·3-1·3) | 2·1 (1·4-3·2) |
| **Sex** | | | | | | | | |
| **Male** | 5·6 (4·5-7·0) | 0·2 (0·1-0·6) | 0·3 (0·1-0·8) | 6·1 (4·9-7·5) | 0·5 (0·2-1·6) | 0·4 (0·1-1·5) | 0·1 (0·0-1·0) | 1·0 (0·4-2·3) |
| **Female** | 5·1 (4·1-6·4) | 1·0 (0·6-1·7) | 0·8 (0·4-1·4) | 6·9 (5·7-8·3) | 0·6 (0·2-1·7) | 1·6 (0·8-3·0) | 1·0 (0·4-2·2) | 3·2 (2·0-5·0) |
| **Age (in years)** | | | | | | | | |
| **18-24** | | | | | | | | |
| **25-29** | | | | | | | | |
| **30-34** | | | | | | | | |
| **35-39** | | | | | | | | |
| **40-44** | | | | | | | | |
| **45-49** | | | | | | | | |
| **50-54** | 8·5 (6·4-11·3) | 0·4 (0·1-1·4) | 0·0 (0·0-0·8) | 8·9 (6·7-11·7) | | | | |
| **55-59** | 4·8 (3·2-7·1) | 0·3 (0·1-1·3) | 0·5 (0·2-1·6) | 5·6 (3·9-8·0) | | | | |
| **60-64** | 3·8 (2·4-6·0) | 0·2 (0·0-1·2) | 0·3 (0·1-1·4) | 4·3 (2·8-6·6) | 0·0 (0·0-1·5) | 0·0 (0·0-1·5) | 0·0 (0·0-1·5) | 0·0 (0·0-1·5) |
| **65-69** | 3·4 (2·0-5·7) | 0·7 (0·2-2·1) | 0·5 (0·1-1·8) | 4·7 (3·0-7·3) | 0·4 (0·1-2·4) | 1·5 (0·5-4·1) | 0·0 (0·0-1·7) | 1·8 (0·7-4·5) |
| **70-74** | 4·1 (2·5-6·6) | 0·9 (0·3-2·5) | 0·5 (0·1-1·9) | 5·6 (3·7-8·4) | 0·4 (0·1-2·5) | 0·7 (0·2-3·0) | 0·8 (0·2-3·2) | 1·9 (0·7-4·8) |
| **75-79** | 4·2 (2·3-7·5) | 1·5 (0·6-3·9) | 1·7 (0·7-4·2) | 7·4 (4·8-11·3) | 0·9 (0·2-3·9) | 0·0 (0·0-2·3) | 0·5 (0·1-3·2) | 1·4 (0·4-4·7) |
| **80-84** | 7·3 (4·3-12·2) | 1·3 (0·4-4·3) | 0·7 (0·1-3·4) | 9·3 (5·8-14·6) | 0·6 (0·1-4·7) | 2·6 (0·8-7·8) | 2·0 (0·6-6·9) | 5·2 (2·3-11·4) |
| **85-89** | 10·0 (5·5-17·4)[c] | 0·0 (0·0-3·7)[c] | 0·8 (0·1-5·1)[c] | 10·8 (6·1-18·3) | 2·9 (0·7-10·8) | 1·0 (0·1-7·9) | 3·0 (0·8-11·0) | 6·9 (2·7-16·3) |
| **90-94** | | | | | 0·0 (0·0-13·8) | 10·7 (3·4-28·8) | 0·0 (0·0-13·8) | 10·7 (3·4-28·8) |
| **95+** | | | | | 0·0 (0·0-39·0) | 0·0 (0·0-39·0) | 0·0 (0·0-39·0) | 0·0 (0·0-39·0) |
| **Working age** | | | | | | | | |
| **Yes** | 5·8 (4·7-7·1) | 0·3 (0·1-0·7) | 0·3 (0·1-0·7) | 6·3 (5·2-7·6) | 0·0 (0·0-1·1) | 0·5 (0·1-1·9) | 0·0 (0·0-1·1) | 0·5 (0·1-1·9) |
| **No** | 4·8 (3·7-6·1) | 1·0 (0·6-1·7) | 0·9 (0·5-1·6) | 6·6 (5·3-8·1) | 0·8 (0·4-1·8) | 1·3 (0·7-2·4) | 0·9 (0·4-1·9) | 3·0 (2·0-4·5) |

**Appendix 5. Continued**

| Strata | Lifelines[a] (n=18,301) | | | |
|---|---|---|---|---|
| | Visual impairment (self-report: A) | Visual impairment (self-report: B) | Visual impairment (self-report: C) | Visual impairment (self-report: D) |
| | Prevalence, % (95% CI) | Prevalence, % (95% CI) | Prevalence, % (95% CI) | Prevalence, % (95% CI) |
| **Total** | 1·0 (0·9-1·2) | 0·4 (0·3-0·5) | 0·2 (0·1-0·3) | 1·6 (1·4-1·8) |
| **Sex** | | | | |
| **Male** | 1·0 (0·8-1·2) | 0·3 (0·2-0·4) | 0·1 (0·1-0·2) | 1·4 (1·2-1·7) |
| **Female** | 1·0 (0·8-1·2) | 0·6 (0·5-0·8) | 0·2 (0·1-0·3) | 1·8 (1·5-2·1) |
| **Age (in years)** | | | | |
| **18-24** | 00·0 (0·0-0·3) | 1·2 (0·8-1·9) | 0·0 (0·0-0·3) | 1·2 (0·8-1·9) |
| **25-29** | 0·0 (0·0-0·3) | 0·0 (0·0-0·3) | 0·0 (0·0-0·3) | 0·0 (0·0-0·3) |
| **30-34** | 0·0 (0·0-0·2) | 0·0 (0·0-0·2) | 0·0 (0·0-0·2) | 0·0 (0·0-0·2) |
| **35-39** | 0·5 (0·2-1·0) | 0·5 (0·2-1·0) | 0·0 (0·0-0·3) | 1·0 (0·6-1·7) |
| **40-44** | 0·4 (0·2-0·9) | 0·6 (0·3-1·2) | 0·0 (0·0-0·3) | 1·0 (0·6-1·7) |
| **45-49** | 0·8 (0·4-1·4) | 0·2 (0·1-0·6) | 0·0 (0·0-0·3) | 1·0 (0·6-1·7) |
| **50-54** | 2·2 (1·6-3·0) | 0·8 (0·5-1·4) | 0·3 (0·1-0·7) | 3·3 (2·5-4·3) |
| **55-59** | 1·4 (0·9-2·1) | 0·5 (0·3-1·0) | 0·2 (0·1-0·6) | 2·0 (1·4-2·8) |
| **60-64** | 1·5 (1·0-2·2) | 0·5 (0·3-1·0) | 0·3 (0·1-0·7) | 2·2 (1·6-3·1) |
| **65-69** | 0·8 (0·4-1·4) | 0·3 (0·1-0·8) | 0·1 (0·0-0·5) | 1·2 (0·7-1·9) |
| **70-74** | 1·1 (0·6-1·9) | 0·4 (0·2-0·9) | 0·4 (0·2-0·9) | 1·8 (1·2-2·7) |
| **75-79** | 1·6 (1·0-2·6) | 0·3 (0·1-0·9) | 0·6 (0·3-1·3) | 2·5 (1·7-3·7) |
| **80-84** | 1·1 (0·5-2·3) | 0·7 (0·3-1·7) | 0·3 (0·1-1·1) | 2·1 (1·2-3·6) |
| **85-89** | 0·9 (0·3-2·6) | 0·0 (0·0-1·1) | 0·9 (0·3-2·6) | 1·9 (0·9-4·0) |
| **90-94** | 17·2 (11·9-24·2)[b] | 0·0 (0·0-2·6)[b] | 0·0 (0·0-2·6)[b] | 17·2 (11·9-24·2)[b] |
| **95+** | | | | |
| **Working age** | | | | |
| **Yes** | 0·8 (0·7-1·0) | 0·5 (0·4-0·6) | 0·1 (0·1-0·2) | 1·3 (1·1-1·5) |
| **No** | 1·6 (1·3-2·0) | 0·4 (0·3-0·6) | 0·4 (0·3-0·6) | 2·4 (2·0-2·9) |

[a] Prevalence of visual impairment based on self-reported data is categorised by four definitions: A: only near vision difficulties; B: only distance vision difficulties; C: both near and distance vision difficulties; D: any vision difficulties

[b] Data is for the age category 90+

[c] Data is for the age category aged 85+

[d] Data is for 10-year intervals: 18-29, 30-39, 40-49, 50-59, 60-69, 70-79, 80-89, 90+

ICPC: International Classification of Primary Care

**Appendix 6. Pooled prevalence estimates of visual impairment from meta-analyses differing in self-report definition, assessment method, and transformation**

Pooled prevalence estimates derived from four separate meta-analyses are presented, each corresponding to a different definition of self-reported visual impairment: (A) near vision problems, (B) distance vision problems, (C) both near and distance vision problems, and (D) any vision problems.

Within each meta-analysis, prevalence estimates are shown separately for three assessment methods: self-report, visual acuity, and ICPC codes; visual acuity and ICPC data are identical across the four meta-analyses; differences in pooled visual acuity and ICPC estimates across the four meta-analyses arise from re-estimation of between-study variance within each model rather than from differences in the underlying data.

Meta-analyses were conducted using random-effects models. Prevalence was estimated using both double arcsine and logit transformations to assess robustness to transformation choice. Reported values represent back-transformed pooled prevalence estimates with 95% confidence intervals.

| Subgroup and transformation | Prevalence (%) | 95% confidence interval |
|---|---|---|
| ***Meta-analysis 1 – self-report near vision problems (A)*** | | |
| Self report double arcsine transformation | 2·04 | 1·21-3·09 |
| Self report logit transformation | 1·81 | 1·01-3·21 |
| Visual acuity double arcsine transformation | 0·30 | 0·00-1·13 |
| Visual acuity logit transformation | 0·28 | 0·11-0·73 |
| ICPC double arcsine transformation | 0·51 | 0·48-0·50 |
| ICPC logit transformation | 0·51 | 0·50-0·52 |
| ***Meta-analysis 2 – self-report distance vision problems (B)*** | | |
| Self report double arcsine transformation | 0·64 | 0·45-0·85 |
| Self report logit transformation | 0·63 | 0·50-0·80 |
| Visual acuity double arcsine transformation | 0·30 | 0·13-0·54 |
| Visual acuity logit transformation | 0·30 | 0·20-0·46 |
| ICPC double arcsine transformation | 0·51 | 0·48-0·50 |
| ICPC logit transformation | 0·51 | 0·50-0·52 |
| ***Meta-analysis 3 – self-report both near and distance vision problems (C)*** | | |
| Self report double arcsine transformation | 0·55 | 0·24-0·98 |
| Self report logit transformation | 0·51 | 0·31-0·83 |
| Visual acuity double arcsine transformation | 0·30 | 0·02-0·86 |
| Visual acuity logit transformation | 0·28 | 0·13-0·62 |
| ICPC double arcsine transformation | 0·51 | 0·48-0·50 |
| ICPC logit transformation | 0·51 | 0·50-0·52 |
| ***Meta-analysis 4 – self-report any vision problems (D)*** | | |
| Self report double arcsine transformation | 3·40 | 2·12-4·97 |
| Self report logit transformation | 3·21 | 2·15-4·77 |
| Visual acuity double arcsine transformation | 0·30 | 0·00-1·35 |
| Visual acuity logit transformation | 0·29 | 0·14-0·58 |
| ICPC double arcsine transformation | 0·51 | 0·48-0·50 |
| ICPC logit transformation | 0·51 | 0·50-0·52 |

ICPC: International Classification of Primary Care

**Appendix 7. Sensitivity analysis: meta-analysis restricted to self-report studies for definition C**

Forest plot showing the results of a sensitivity analysis restricted to self-report datasets for definition C (self-reported difficulties with both near and distance vision). This analysis was performed to assess whether the inclusion of visual acuity and ICPC datasets in the primary meta-analyses influenced the pooled prevalence estimates.

The meta-analysis was repeated using only the self-report datasets corresponding to definition C, excluding visual acuity and ICPC datasets. The same statistical approach as in the primary analyses was used: random-effects meta-analysis of proportions with logit transformation and DerSimonian-Laird estimation of between-study variance. Study-specific prevalence estimates and their 95% confidence intervals are shown, along with the pooled prevalence estimate.

Results can be compared with the corresponding pooled estimate from the primary joint analysis (0·51% [95% CI 0·31-0·83]), which included self-report, visual acuity, and ICPC datasets. The similar pooled estimate obtained in this sensitivity analysis (0·50% [0·28-0·89]) indicates that inclusion of visual acuity and ICPC datasets does not materially influence the pooled prevalence estimates.

| Study | Cases | Total | Prevalence | 95% CI |
|---|---|---|---|---|
| GEMON 2020 | 5035 | 514967 | 0.98 | [0.95; 1.00] |
| GECON 2020-2022 | 156 | 21917 | 0.71 | [0.60; 0.83] |
| Lifelines 2021-2024 | 30 | 18301 | 0.16 | [0.11; 0.23] |
| Doetinchem 2018-2022 | 14 | 2731 | 0.51 | [0.28; 0.86] |
| LASA 2021-2022 | 6 | 1047 | 0.57 | [0.21; 1.24] |
| **Random effects model** | | | **0.50** | **[0.28; 0.89]** |

Heterogeneity: $I^2 = 96.6\%$, $\tau^2 = 0.3772$, $\chi^2_4 = 117.32$ ($p < 0.0001$)

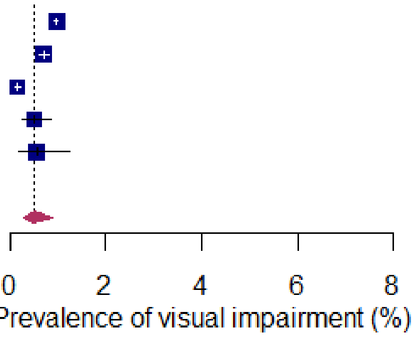

**Appendix 8. Estimated number of adults with visual impairment in the Netherlands (2025-2050)**

Estimates were obtained by applying age-specific prevalence estimates from the meta-analyses to population projections from Statistics Netherlands. Results are shown separately for each assessment method (visual acuity, ICPC codes and self-report according to four definitions). Estimates reflect modelled prevalence rather than directly observed counts.

| | 2025 | 2030 | 2040 | 2050 |
|---|---|---|---|---|
| Assessment method[a] | Cases, n (95% CI) | Cases, n (95% CI) | Cases, n (95% CI) | Cases, n (95% CI) |
| Visual acuity | 39,132 (17,716-95,749) | 41,553 (18,822-101,574) | 45,864 (20,808-111,809) | 48,823 (22,182-118,759) |
| ICPC codes | 75,719 (33,294-189,032) | 80,347 (35,356-200,324) | 88,513 (39,036-219,898) | 94,079 (41,568-233,058) |
| Self-report: A | 228,483 (66,737-755,160) | 239,678 (70,027-791,411) | 256,428 (74,957-845,395) | 266,570 (77,940-878,133) |
| Self-report: B | 87,534 (60,660-126,169) | 92,992 (64,449-134,014) | 102,886 (71,325-148,210) | 109,833 (76,154-158,166) |
| Self-report: C | 66,320 (24,246-179,730) | 71,332 (26,086-193,154) | 81,232 (29,737-219,363) | 88,440 (32,403-238,278) |
| Self-report: D | 406,350 (167,495-956,615) | 428,911 (176,924-1,008,087) | 465,732 (192,434-1,090,632) | 489,099 (202,328-1,142,432) |

[a] Prevalence of visual impairment based on self-reported data is categorised by four definitions: A: only near vision difficulties; B: only distance vision difficulties; C: both near and distance vision difficulties; D: any vision difficulties

ICPC: International Classification of Primary Care

**Appendix 9. Stratified projected number of adults with visual impairment by age group and assessment method (2025-2050)**

Projections were based on age-specific prevalence estimates derived from logistic mixed-effects models and applied to population projections. Results are shown separately for each assessment method (visual acuity, ICPC codes and self-report according to four definitions). Estimates reflect modelled prevalence under the assumption of constant age-specific rates over time.

| Age group | 2025 Cases, n (95% CI) | 2030 Cases, n (95% CI) | 2040 Cases, n (95% CI) | 2050 Cases, n (95% CI) |
|---|---|---|---|---|
| **Visual acuity** | | | | |
| 18+ | 39,132 (17,715–95,748) | 41,552 (18,821–101,573) | 45,864 (20,808–111,809) | 48,822 (22,182–118,758) |
| 18-24 | 2,166 (1,102–4,749) | 2,095 (1,065–4,592) | 1,987 (1,011–4,357) | 2,006 (1,020–4,398) |
| 25-29 | 1,635 (810–3,685) | 1,726 (856–3,890) | 1,613 (800–3,635) | 1,558 (772–3,511) |
| 30-34 | 1,756 (846–4,069) | 1,802 (869–4,176) | 1,805 (870–4,183) | 1,723 (831–3,993) |
| 35-39 | 1,779 (834–4,238) | 1,923 (901–4,580) | 2,036 (955–4,850) | 1,914 (897–4,558) |
| 40-44 | 1,810 (826–4,419) | 1,958 (894–4,781) | 2,127 (971–5,195) | 2,124 (970–5,187) |
| 45-49 | 1,933 (862–4,820) | 2,018 (900–5,031) | 2,311 (1,031–5,762) | 2,438 (1,087–6,079) |
| 50-54 | 2,489 (1,090–6,302) | 2,185 (957–5,532) | 2,425 (1,062–6,138) | 2,621 (1,148–6,634) |
| 55-59 | 3,044 (1,318–7,775) | 2,816 (1,220–7,193) | 2,555 (1,107–6,526) | 2,910 (1,260–7,432) |
| 60-64 | 3,428 (1,480–8,770) | 3,462 (1,494–8,856) | 2,805 (1,211–7,176) | 3,105 (1,340–7,944) |
| 65-69 | 3,583 (1,554–9,113) | 3,912 (1,697–9,949) | 3,671 (1,592–9,335) | 3,335 (1,446–8,482) |
| 70-74 | 3,785 (1,660–9,509) | 4,092 (1,795–10,281) | 4,575 (2,007–11,495) | 3,740 (1,640–9,396) |
| 75-79 | 4,254 (1,895–10,511) | 4,233 (1,886–10,460) | 5,129 (2,285–12,674) | 4,924 (2,194–12,168) |
| 80-84 | 3,319 (1,505–8,045) | 4,461 (2,023–10,811) | 5,052 (2,290–12,242) | 5,891 (2,671–14,278) |
| 85-89 | 2,449 (1,129–5,814) | 2,940 (1,356–6,981) | 4,243 (1,957–10,073) | 5,597 (2,581–13,288) |
| 90-94 | 1,310 (613–3,047) | 1,507 (705–3,505) | 2,771 (1,297–6,444) | 3,547 (1,661–8,251) |
| 95+ | 383 (181–876) | 414 (196–946) | 752 (356–1,717) | 1,382 (654–3,154) |
| **ICPC** | | | | |
| 18+ | 75,719 (33,294–189,032) | 80,346 (35,356–200,323) | 88,513 (39,036–219,897) | 94,079 (41,568–233,058) |
| 18-24 | 4,040 (1,999–9,095) | 3,907 (1,933–8,795) | 3,707 (1,834–8,344) | 3,742 (1,852–8,423) |
| 25-29 | 3,082 (1,484–7,133) | 3,253 (1,566–7,530) | 3,040 (1,464–7,036) | 2,936 (1,413–6,796) |
| 30-34 | 3,344 (1,564–7,961) | 3,432 (1,605–8,169) | 3,437 (1,607–8,182) | 3,281 (1,534–7,811) |
| 35-39 | 3,423 (1,555–8,372) | 3,700 (1,681–9,049) | 3,918 (1,780–9,582) | 3,683 (1,673–9,006) |
| 40-44 | 3,515 (1,555–8,805) | 3,803 (1,682–9,524) | 4,132 (1,827–10,349) | 4,126 (1,824–10,333) |
| 45-49 | 3,784 (1,635–9,664) | 3,950 (1,707–10,088) | 4,524 (1,955–11,553) | 4,773 (2,062–12,189) |
| 50-54 | 4,900 (2,080–12,688) | 4,301 (1,826–11,137) | 4,773 (2,026–12,358) | 5,158 (2,190–13,356) |
| 55-59 | 6,009 (2,524–15,675) | 5,559 (2,335–14,502) | 5,044 (2,118–13,157) | 5,744 (2,412–14,984) |
| 60-64 | 6,766 (2,834–17,655) | 6,832 (2,862–17,829) | 5,536 (2,319–14,445) | 6,129 (2,567–15,993) |
| 65-69 | 7,047 (2,968–18,261) | 7,694 (3,241–19,937) | 7,219 (3,040–18,705) | 6,559 (2,762–16,996) |
| 70-74 | 7,395 (3,153–18,911) | 7,996 (3,409–20,446) | 8,940 (3,812–22,861) | 7,307 (3,116–18,685) |
| 75-79 | 8,240 (3,573–20,691) | 8,200 (3,556–20,591) | 9,935 (4,309–24,949) | 9,539 (4,137–23,953) |
| 80-84 | 6,363 (2,813–15,637) | 8,552 (3,781–21,015) | 9,684 (4,281–23,796) | 11,293 (4,993–27,752) |
| 85-89 | 4,641 (2,092–11,132) | 5,572 (2,512–13,366) | 8,040 (3,625–19,285) | 10,605 (4,782–25,439) |
| 90-94 | 2,452 (1,126–5,729) | 2,821 (1,296–6,591) | 5,187 (2,382–12,118) | 6,641 (3,050–15,516) |
| 95+ | 710 (331–1,616) | 767 (357–1,746) | 1,392 (649–3,169) | 2,557 (1,192–5,820) |
| **Self-report: A[a]** | | | | |
| 18+ | 228,482 (66,736–755,160) | 239,677 (70,027–791,410) | 256,427 (74,957–845,394) | 266,570 (77,940–878,133) |
| 18-24 | 6,115 (1,762–21,077) | 5,914 (1,704–20,383) | 5,610 (1,617–19,337) | 5,664 (1,632–19,520) |
| 25-29 | 5,827 (1,681–20,021) | 6,151 (1,775–21,136) | 5,749 (1,659–19,751) | 5,552 (1,602–19,076) |
| 30-34 | 7,730 (2,233–26,461) | 7,933 (2,292–27,155) | 7,946 (2,295–27,198) | 7,585 (2,191–25,963) |
| 35-39 | 9,412 (2,723–32,074) | 10,173 (2,943–34,667) | 10,772 (3,116–36,707) | 10,124 (2,929–34,501) |
| 40-44 | 11,136 (3,227–37,750) | 12,046 (3,491–40,835) | 13,089 (3,793–44,371) | 13,069 (3,787–44,302) |
| 45-49 | 13,352 (3,876–44,988) | 13,937 (4,046–46,961) | 15,961 (4,634–53,780) | 16,839 (4,889–56,739) |
| 50-54 | 18,585 (5,407–62,199) | 16,314 (4,746–54,600) | 18,103 (5,267–60,585) | 19,565 (5,692–65,478) |
| 55-59 | 23,611 (6,884–78,453) | 21,845 (6,369–72,585) | 19,819 (5,778–65,851) | 22,570 (6,581–74,994) |
| 60-64 | 26,462 (7,733–87,287) | 26,722 (7,809–88,144) | 21,650 (6,327–71,416) | 23,969 (7,004–79,065) |
| 65-69 | 26,257 (7,689–86,021) | 28,666 (8,395–93,914) | 26,896 (7,876–88,115) | 24,438 (7,156–80,060) |
| 70-74 | 25,153 (7,380–81,901) | 27,195 (7,979–88,549) | 30,407 (8,922–99,009) | 24,854 (7,292–80,926) |
| 75-79 | 24,552 (7,216–79,517) | 24,434 (7,181–79,135) | 29,604 (8,700–95,880) | 28,422 (8,353–92,053) |
| 80-84 | 15,970 (4,699–51,488) | 21,461 (6,316–69,194) | 24,302 (7,152–78,352) | 28,342 (8,341–91,377) |
| 85-89 | 9,462 (2,787–30,392) | 11,362 (3,347–36,492) | 16,393 (4,829–52,652) | 21,624 (6,370–69,454) |
| 90-94 | 3,944 (1,162–12,627) | 4,537 (1,337–14,526) | 8,342 (2,459–26,706) | 10,681 (3,149–34,193) |
| 95+ | 907 (267–2,895) | 979 (288–3,127) | 1,777 (524–5,675) | 3,265 (963–10,424) |

| | | | | |
|---|---|---|---|---|
| **Self-report: B[a]** | | | | |
| 18+ | 87,534 (60,659–126,169) | 92,991 (64,448–134,013) | 102,886 (71,325–148,209) | 109,833 (76,154–158,165) |
| 18-24 | 7,442 (5,142–10,763) | 7,197 (4,973–10,409) | 6,828 (4,718–9,875) | 6,892 (4,762–9,968) |
| 25-29 | 5,153 (3,564–7,444) | 5,439 (3,762–7,859) | 5,083 (3,516–7,344) | 4,909 (3,396–7,093) |
| 30-34 | 5,030 (3,481–7,262) | 5,161 (3,573–7,453) | 5,170 (3,578–7,464) | 4,935 (3,416–7,125) |
| 35-39 | 4,611 (3,192–6,655) | 4,983 (3,450–7,193) | 5,277 (3,654–7,617) | 4,960 (3,434–7,159) |
| 40-44 | 4,246 (2,940–6,129) | 4,593 (3,181–6,630) | 4,991 (3,456–7,204) | 4,983 (3,451–7,193) |
| 45-49 | 4,140 (2,866–5,975) | 4,321 (2,992–6,237) | 4,949 (3,426–7,143) | 5,221 (3,615–7,536) |
| 50-54 | 4,944 (3,423–7,137) | 4,340 (3,005–6,265) | 4,816 (3,334–6,952) | 5,205 (3,604–7,513) |
| 55-59 | 5,746 (3,978–8,293) | 5,316 (3,681–7,673) | 4,823 (3,339–6,961) | 5,492 (3,803–7,927) |
| 60-64 | 6,343 (4,393–9,151) | 6,405 (4,436–9,241) | 5,189 (3,594–7,487) | 5,745 (3,979–8,289) |
| 65-69 | 6,717 (4,654–9,685) | 7,333 (5,082–10,573) | 6,880 (4,768–9,920) | 6,251 (4,332–9,014) |
| 70-74 | 7,376 (5,114–10,626) | 7,974 (5,529–11,488) | 8,916 (6,183–12,845) | 7,288 (5,053–10,499) |
| 75-79 | 8,753 (6,073–12,596) | 8,711 (6,044–12,536) | 10,554 (7,323–15,189) | 10,133 (7,031–14,582) |
| 80-84 | 7,249 (5,033–10,420) | 9,742 (6,764–14,004) | 11,031 (7,659–15,857) | 12,865 (8,932–18,493) |
| 85-89 | 5,655 (3,929–8,118) | 6,790 (4,717–9,747) | 9,797 (6,806–14,064) | 12,924 (8,978–18,552) |
| 90-94 | 3,167 (2,202–4,539) | 3,644 (2,533–5,221) | 6,700 (4,658–9,600) | 8,578 (5,964–12,291) |
| 95+ | 956 (665–1,367) | 1,033 (719–1,476) | 1,875 (1,305–2,680) | 3,444 (2,397–4,922) |
| **Self-report: C[a]** | | | | |
| 18+ | 66,320 (24,245–179,730) | 71,332 (26,086–193,154) | 81,232 (29,736–219,362) | 88,440 (32,403–238,277) |
| 18-24 | 3,724 (1,354–10,212) | 3,601 (1,310–9,876) | 3,416 (1,242–9,369) | 3,449 (1,254–9,458) |
| 25-29 | 2,724 (991–7,466) | 2,875 (1,046–7,881) | 2,687 (978–7,365) | 2,595 (944–7,113) |
| 30-34 | 2,809 (1,022–7,696) | 2,882 (1,049–7,898) | 2,887 (1,051–7,910) | 2,756 (1,003–7,551) |
| 35-39 | 2,719 (990–7,449) | 2,939 (1,070–8,052) | 3,112 (1,133–8,526) | 2,925 (1,065–8,013) |
| 40-44 | 2,645 (963–7,243) | 2,861 (1,042–7,835) | 3,109 (1,132–8,513) | 3,104 (1,130–8,500) |
| 45-49 | 2,722 (991–7,451) | 2,841 (1,034–7,778) | 3,254 (1,185–8,908) | 3,433 (1,250–9,398) |
| 50-54 | 3,430 (1,249–9,386) | 3,011 (1,096–8,239) | 3,341 (1,217–9,143) | 3,611 (1,315–9,881) |
| 55-59 | 4,204 (1,531–11,495) | 3,889 (1,417–10,635) | 3,528 (1,285–9,648) | 4,018 (1,464–10,988) |
| 60-64 | 4,891 (1,783–13,357) | 4,939 (1,800–13,489) | 4,002 (1,458–10,929) | 4,430 (1,615–12,099) |
| 65-69 | 5,455 (1,990–14,869) | 5,956 (2,173–16,234) | 5,588 (2,038–15,231) | 5,077 (1,852–13,839) |
| 70-74 | 6,305 (2,303–17,135) | 6,817 (2,490–18,526) | 7,623 (2,784–20,714) | 6,230 (2,275–16,931) |
| 75-79 | 7,872 (2,880–21,293) | 7,834 (2,866–21,190) | 9,491 (3,472–25,675) | 9,112 (3,334–24,650) |
| 80-84 | 6,854 (2,514–18,413) | 9,211 (3,378–24,745) | 10,430 (3,825–28,021) | 12,164 (4,461–32,679) |
| 85-89 | 5,617 (2,068–14,937) | 6,745 (2,483–17,935) | 9,732 (3,582–25,877) | 12,838 (4,726–34,134) |
| 90-94 | 3,303 (1,222–8,648) | 3,799 (1,406–9,949) | 6,986 (2,586–18,291) | 8,944 (3,311–23,419) |
| 95+ | 1,040 (388–2,671) | 1,124 (419–2,885) | 2,040 (760–5,236) | 3,747 (1,397–9,617) |
| **Self-report: D[a]** | | | | |
| 18+ | 406,349 (167,494–956,615) | 428,911 (176,923–1,008,086) | 465,731 (192,434–1,090,631) | 489,098 (202,328–1,142,431) |
| 18-24 | 18,480 (7,496–45,106) | 17,872 (7,249–43,622) | 16,954 (6,877–41,382) | 17,115 (6,942–41,774) |
| 25-29 | 15,315 (6,219–37,293) | 16,167 (6,565–39,369) | 15,108 (6,135–36,790) | 14,592 (5,925–35,532) |
| 30-34 | 17,768 (7,224–43,158) | 18,234 (7,413–44,289) | 18,263 (7,425–44,360) | 17,434 (7,087–42,345) |
| 35-39 | 19,114 (7,780–46,296) | 20,659 (8,409–50,038) | 21,875 (8,904–52,984) | 20,561 (8,369–49,799) |
| 40-44 | 20,278 (8,265–48,958) | 21,935 (8,941–52,959) | 23,835 (9,715–57,545) | 23,797 (9,700–57,455) |
| 45-49 | 22,224 (9,073–53,457) | 23,198 (9,470–55,801) | 26,567 (10,846–63,904) | 28,029 (11,442–67,420) |
| 50-54 | 28,953 (11,842–69,342) | 25,416 (10,395–60,870) | 28,202 (11,534–67,543) | 30,480 (12,466–72,998) |
| 55-59 | 35,407 (14,513–84,357) | 32,758 (13,428–78,047) | 29,719 (12,182–70,807) | 33,846 (13,873–80,638) |
| 60-64 | 39,446 (16,213–93,393) | 39,834 (16,372–94,311) | 32,274 (13,265–76,412) | 35,731 (14,686–84,596) |
| 65-69 | 40,283 (16,612–94,649) | 43,980 (18,136–103,334) | 41,264 (17,016–96,953) | 37,492 (15,461–88,091) |
| 70-74 | 40,951 (16,955–95,333) | 44,275 (18,332–103,072) | 49,504 (20,497–115,246) | 40,463 (16,754–94,199) |
| 75-79 | 43,483 (18,092–100,117) | 43,273 (18,005–99,635) | 52,430 (21,815–120,719) | 50,337 (20,944–115,899) |
| 80-84 | 31,361 (13,125–71,269) | 42,146 (17,639–95,776) | 47,724 (19,974–108,452) | 55,657 (23,294–126,481) |
| 85-89 | 20,883 (8,801–46,731) | 25,074 (10,568–56,110) | 36,177 (15,248–80,957) | 47,722 (20,113–106,791) |
| 90-94 | 9,857 (4,189–21,667) | 11,340 (4,819–24,927) | 20,848 (8,860–45,827) | 26,693 (11,344–58,674) |
| 95+ | 2,539 (1,088–5,480) | 2,742 (1,175–5,919) | 4,978 (2,134–10,742) | 9,143 (3,919–19,731) |

[a] Prevalence of visual impairment based on self-reported data is categorized by four definitions: A: only near vision difficulties; B: only distance vision difficulties; C: both near and distance vision difficulties; D: any vision difficulties
ICPC: International Classification of Primary Care
