# Supplementary material for: Estimating the prevalence of visual impairment in the Netherlands, with forecasts up to 2050: a meta-analysis of national databases
Source: eClinicalMedicine. 2026 Apr 1;94:103858. doi: 10.1016/j.eclinm.2026.103858 (PMC13087707; doi:10.1016/j.eclinm.2026.103858)
Supplement: Translated Abstract [file mmc2.docx]

*The following translations in Dutch were submitted by the authors and we reproduce them as supplied. They have not been peer reviewed. Our editorial processes have only been applied to the original abstract in English, which should serve as reference for this manuscript.*

**Nederlandse samenvatting**

**Achtergrond:** Recente prevalentieschattingen van visuele beperkingen in Nederland ontbreken. Op basis van representatieve databanken schat deze studie de prevalentie van visuele beperkingen onder Nederlandse volwassenen en worden prognoses tot 2050 gemaakt.

**Methoden:** Databanken werden voornamelijk geïdentificeerd via consultatie van experts en geïncludeerd indien zij representatief waren voor de Nederlandse bevolking en recente (≥2010) gegevens bevatten over zicht (gezichtsscherpte <6/18, zelf-gerapporteerd visueel functioneren of relevante ICPC-codes). Zelf-gerapporteerd visueel functioneren werd geclassificeerd volgens vier definities: A) alleen problemen met nabij zien; B) alleen problemen met veraf zien; C) zowel problemen met nabij als met veraf zien; D) enige vorm van visuele problemen (combinatie van A-C). Prevalenties werden berekend met behulp van vier meta-analyses, inclusief heterogeniteitsanalyses en subgroep analyses. Leeftijdseffecten werden gemodelleerd met logistische mixed-effectmodellen.

**Resultaten:** Acht databanken met gegevens verzameld tussen 2010 en 2024 werden geïncludeerd (n = 1.814.716; 894.541 mannen / 920.175 vrouwen). Vijf databanken betroffen prospectieve, populatie gebaseerde cohortstudies, twee bestonden uit periodiek uitgevoerde gezondheidsenquêtes en één bevatte registraties uit huisartsenpraktijken. Vijf databanken bevatten zelf-gerapporteerde gegevens over visueel functioneren, twee bevatten gegevens over best-gecorrigeerde gezichtsscherpte en één bevatte ICPC-codes. Er was sprake van substantiële heterogeniteit, waarbij de meetmethode als moderator fungeerde (zelfrapportage versus gezichtsscherpte versus ICPC; I²-statistiek >98%, p<0,0001). De gepoolde prevalentie van visuele beperkingen bedroeg 0,28% (95%BI: 0,11–0,73) op basis van visusmetingen (2 databanken), en 0,51% (95%BI: 0,50–0,52) op basis van ICPC-codes (1 databank). Voor zelfrapportage (vijf databanken) was de prevalentie afhankelijk van de definitie: voor alleen problemen met nabij zien (A) was de prevalentie 1,81% (95%BI: 1,01–3,21); voor alleen problemen met veraf zien (B) 0,63% (95%BI: 0,50–0,80); voor zowel problemen met nabij als veraf zien (C) 0,51% (95%BI: 0,31–0,83); en voor enige vorm van visuele problemen (D) 3,21% (95%BI: 2,15–4,77). Na toepassing van leeftijds- en meetmethode-specifieke prevalenties op geprojecteerde bevolkingsstructuren wordt geschat dat in 2025 tussen de 39.100 en 406.400 Nederlandse volwassenen een visuele beperking hebben, oplopend tot 48.800–489.100 in 2050 als gevolg van bevolkingsgroei en vergrijzing, afhankelijk van de gebruikte definitie.

**Interpretatie:** Ondanks inherente beperkingen, waaronder het overwegend gebruik van zelf-gerapporteerde gegevens over visueel functioneren en de aanzienlijke onzekerheid in de schattingen, biedt deze studie schattingen van de prevalentie van visuele beperkingen in Nederland. Hoewel de schattingen een brede bandbreedte kennen, laten zij duidelijke trends en plausibele onzekerheidsintervallen zien en bieden zij inzicht in de huidige en toekomstige ziektelast van visuele beperkingen.

**Financiering:** Oogfonds.
